# Empowering women through trauma-informed maternity care: the EMPATHY framework

**DOI:** 10.3389/fgwh.2025.1608174

**Published:** 2025-12-04

**Authors:** Joanne Cull, Gill Thomson, Soo Downe, Anastasia Topalidou, Michelle Fine

**Affiliations:** 1 School of Nursing and Midwifery, London South Bank University, London, United Kingdom; 2 School of Community Health and Midwifery, The University of Lancashire, Preston, England, United Kingdom; 3 Public Science Project, The Graduate Center, The City University of New York, New York, NY, United States

**Keywords:** trauma-Informed care (TIC), perinatal mental health, participatory research (PR), maternity care, patient empowerment, adverse childhood experience (ACE)

## Abstract

**Background:**

At least one in four women in the UK has experienced trauma, such as sexual abuse or violence, with profound implications for mental and physical health, particularly during the perinatal period. Despite the potential benefits of addressing trauma in maternity care, many women are reluctant to disclose their experiences due to stigma, fear of judgment, or lack of trust in healthcare systems. This paper presents the development and evaluation of the EMPATHY framework, a novel, evidence-based approach to routine trauma discussions in maternity care, designed to address these challenges and promote emotionally-centred care.

**Methods:**

The EMPATHY framework was developed through a critical participatory action research approach, integrating findings from a systematic review, qualitative interviews, and stakeholder input, including experts by experience, healthcare professionals, and voluntary sector practitioners. The framework was refined through iterative workshops and a public consultation (*n* = 52), ensuring its relevance and applicability. The development and evaluation of the EMPATHY framework were guided by the Appraisal of Guidelines for Research and Evaluation II (AGREE II) tool, ensuring methodological rigor, transparency, and adherence to established standards in guideline development.

**Results:**

The framework is structured around six core principles: system-wide change, promote trauma awareness, trust and relationships, training and support, local tailoring, and continuous improvement. A key innovation is the recommendation that all women, regardless of disclosure, should have access to information and support. Feedback from the public consultation highlighted the framework's value and its potential to transform perinatal experiences. Challenges such as resource constraints and implementation barriers were acknowledged, but respondents emphasised the importance of the framework in improving care for women who have experienced trauma.

**Discussion/conclusion:**

The EMPATHY framework addresses a critical gap in existing guidance by offering a structured yet flexible approach to routine trauma discussions. Its implementation has the potential to empower women, strengthen therapeutic relationships, and reduce re-traumatisation. The framework represents a significant step forward in trauma-informed perinatal care.

## Introduction

At least one in four women in the United Kingdom has experienced trauma, such as sexual abuse or violence, with profound implications for mental and physical health, well-being, and interpersonal relationships (1, 2). Large-scale population studies in England and Wales show that childhood trauma is cumulatively associated with physical and mental health risks, including substance use, elevated body mass index, cardiovascular disease, and mental illness (2). Trauma can influence pregnancy outcomes through both physiological and behavioural pathways, with repeated exposures increasing risk (3).

The perinatal period, which is marked by significant physical and emotional changes, can exacerbate the effects of trauma (4). For some women, the physiological changes of pregnancy may trigger flashbacks or lead them to ruminate on their own childhood experiences as they contemplate parenthood (5). Trauma is closely linked to mental health challenges, including maternal suicide, which remains a leading cause of maternal mortality (6). The intimate nature of maternity care procedures, coupled with the potential for new or worsening mental health challenges, underscores the need for sensitive and effective support during this critical time (7).

Pregnancy is a powerful time to offer support to women affected by trauma (8). Women are often motivated to improve their health and well-being for the sake of their unborn child, and have frequent contact with healthcare providers in the perinatal period. They frequently engage with healthcare providers, particularly midwives, who are uniquely positioned as trusted professionals (9). However, despite the potential benefits, women rarely disclose previous trauma without prompting due to strong social taboos and the stigma surrounding disclosure (5). This reluctance highlights the need for a structured, compassionate approach to trauma conversations within maternity care.

### The case for routine trauma discussions

Embedding discussions of previous trauma as a routine component of maternity care, rather than on the basis of clinician concern about individual women, is essential to mitigate clinician bias and ensure equitable care (10). Evidence suggests that both women and clinicians find such discussions valuable and worthwhile (21). Without routine trauma discussions, care providers may miss critical opportunities to support women in distress. Furthermore, even when women choose not to disclose at this time, sensitively raising the issue prepares them for the emotional challenges of the perinatal period and may facilitate future disclosure (37).

However, initiating trauma discussions is not without challenges. Raising the issue insensitively or without adequate forewarning can be futile or even harmful (11). Women may find such conversations unexpected, intrusive, or distressing, potentially leading to disengagement from maternity services (12). Overzealous safeguarding responses or unwarranted referrals to safeguarding or mental health services can further alienate women, while the lack of trauma-informed support services often leaves clinicians ill-equipped to respond effectively (21). These complexities underscore the need for a carefully designed framework to guide trauma conversations in maternity care.

### Challenges in current practice

Existing tools and approaches for trauma discussions often fall short. Commonly used instruments, such as the Adverse Childhood Experiences (ACE) score, have been criticised for their potential to harm the clinician-patient relationship and their limited effectiveness in identifying and addressing trauma-related needs (13). Asking patients to complete an ACE questionnaire can trigger shame, embarrassment, or painful memories of past trauma, particularly if administered without adequate support. Pregnant women may worry about the impact of their experiences on their unborn child, which can increase anxiety and feelings of disempowerment (32). Furthermore, ACE scores were designed for population-level research rather than individual risk prediction, and relying on these scores in clinical decision-making can oversimplify complex experiences and inadvertently pathologise patients (33, 34). Standardised questionnaires may also fail to capture protective factors or the socio-political context of trauma, and they can further marginalise vulnerable groups, such as people with low literacy, limited English proficiency, or cognitive differences (21, 35). The EMPATHY study emphasises a woman-centred, compassionate approach that prioritises open communication and empathy, creating a safer environment for discussing previous trauma while minimising potential harms.

Clinicians face significant challenges in conducting trauma discussions. Women who have experienced trauma may exhibit heightened distress, fear, or frustration during perinatal care, which can occasionally manifest as challenging behaviours (14). These behaviours are best understood as responses to past trauma rather than intrinsic traits, underscoring the importance of trauma-informed approaches that prioritise empathy, trust-building, and safety. Hearing distressing disclosures can also evoke personal memories of trauma among care providers, highlighting the need for reflexive supervision and support (15). Without such infrastructure, trauma discussions risk re-traumatising both women and clinicians, complicating the delivery of compassionate care.

### The need for a new framework

Given these challenges, there is a pressing need for a structured, woman-centred framework to guide trauma conversations in maternity care. Such a framework should account for the timing, setting, and methodology of discussions, as well as the training and support needs of clinicians (31). It must also prioritise cultural acceptability and accessibility, particularly for vulnerable populations such as ethnic minorities and socially excluded groups, who are disproportionately affected by trauma, yet less likely to access support (16).

This paper introduces the development and evaluation of the EMPATHY framework, a fresh approach to facilitating discussions about previous trauma in maternity care. Designed to address the limitations of existing practices, the framework promotes equitable, compassionate, and emotionally-centered care. By integrating insights from a systematic review of trauma discussions in maternity care, interview findings, and expert input from lived and professional experience, the framework aims to facilitate meaningful discussions, support women in distress, and ultimately interrupt the intergenerational transmission of trauma.

## Methods

### Reflexive note

In developing the EMPATHY framework, we critically reflected on our pre-existing beliefs about routine trauma discussions and how these might influence the design and implementation of the framework.

The research team brought diverse perspectives to the project. JC, a midwife and doctoral student, was uncertain about the benefits of routine trauma discussions, particularly for women facing discrimination based on factors such as race, class, or immigration status. She was concerned that disclosure could lead to unnecessary safeguarding interventions or mental health referrals, potentially causing harm rather than providing support. SD, a midwife with 18 years of clinical experience and a background in maternity care research, shared similar concerns about the potential risks of routine trauma discussions. GT, a maternity care researcher with a psychology background and extensive experience in perinatal mental health research, emphasised the importance of trauma-informed conversations to enable needs-led care. AT, a maternal and neonatal care researcher, highlighted the necessity of a supportive care model to facilitate meaningful trauma discussions. MF, a critical psychology scholar with expertise in participatory work with marginalised communities, contributed insights into trauma as both a source of pain and a site of resilience, knowledge, and activism.

### The EMPATHY study

The framework was created as the concluding element of the EMPATHY (EMpowering Pregnant women Affected by Trauma HistorY) study, a doctoral project grounded in critical participatory action research (21).

This study was conducted within the UK National Health Service (NHS), where maternity care is publicly funded, universally accessible and primarily delivered by midwives, with escalation to obstetric or mental health services when required. Continuity of care is implemented inconsistently across regions. Although routine perinatal mental health screening is recommended in national policy, routine enquiry about previous trauma is not currently included.

The study was guided by a Research Collective, a group of 18 women which included women with trauma histories, voluntary sector practitioners, and healthcare professionals. The Research Collective first met prior to the doctoral funding application, shaping the study's design and conceptualisation from the outset. They played a central role, providing feedback on the study design, interview methods, and the development of the EMPATHY framework. Across six workshops (five online, one in-person), they offered insights on stakeholder engagement, interview guides, and dissemination strategies. Their contributions ensured the study remained inclusive, equitable, and grounded in real-world perspectives.

Phases of the study are shown in Table 1. A systematic literature review and qualitative evidence synthesis were conducted, incorporating 25 papers from five countries, which included perspectives from 1,602 women and 286 healthcare professionals and voluntary sector experts (21). The review, conducted in July 2021 and updated in April 2022, included 25 papers from five high-income countries published between 2001 and 2022. Study quality was assessed using the Critical Appraisal Skills Programme (CASP) checklist, and findings were thematically synthesised. Confidence in the evidence was evaluated using the GRADE-CERQual approach, with most findings rated as moderate or high.

**Table 1:** Phases of the EMPATHY study.

Study phase	Participant numbers
Systematic review and qualitative synthesis (21)	25 papers from 5 countries included, representing the views of 1,602 women and 286 healthcare professionals and experts from the voluntary sector
Interviews (37)	Women with trauma histories (*n* = 4), healthcare professionals (*n* = 12), and voluntary sector experts (*n* = 7)
Public consultation on framework (described in this paper)	52 respondents to the consultation

Semi-structured interviews were conducted with key stakeholders, including experts by experience (*n* = 4), voluntary sector representatives (*n* = 7), and healthcare providers (*n* = 12). Reflexive thematic analysis was used to explore participants’ perspectives on the acceptability, feasibility, and value of routine trauma discussions in maternity care (37).

Findings from the qualitative synthesis and interviews were analysed independently and then combined with input from the Research Collective to formulate an evidence-based framework of guiding principles for discussing previous trauma during the perinatal period. The framework's development also involved a rigorous public consultation, which received 52 responses. The development and evaluation of the framework are described in this paper.

### Development of the framework

Findings from the review and interviews identified that effective and sensitive trauma discussions require more than just an appropriate tool or methodology; they also need an environment conducive to disclosure. Key elements include addressing concerns about confidentiality, providing sufficient time and context for discussions, and developing trusting relationships. The study also highlighted the need to critically examine assumptions about the benefits of trauma discussions and to assess their acceptability and utility. Practitioner-level data and interview findings revealed that trauma discussions were often incorporated into care providers’ responsibilities without adequate training, resources, or support. Consequently, it was deemed essential to develop a broad-ranging, foundational set of guiding principles outlining all aspects of effective and sensitive trauma discussions.

The framework was informed by the systematic review and qualitative synthesis of existing literature on routine trauma discussions and interviews referred to above (21, 37). In addition, the framework incorporated guidance from seminal documents on trauma-informed care including SAMHSA's *Concept of Trauma and Guidance for a Trauma-Informed Approach* (17) and NHS England's *Good Practice Guide to Implementing Trauma-Informed Care in the Perinatal Period* (18).

### Development process and AGREE II guidance

The framework was developed and evaluated in accordance with the Appraisal of Guidelines for Research and Evaluation II (AGREE II) tool, a widely accepted standard for clinical practice guidelines (19, 20). Table 2 presents the six quality domains of AGREE II and how they were addressed in the study.

**Table 2:** Application of AGREE II quality domains in the development of the EMPATHY framework.

AGREE II domain	Description	How the domain was addressed in the EMPATHY framework
Scope and purpose	Clearly define the aim, health questions, and target population.	The framework aims to: 1. Provide guidance on sensitive and effective trauma discussions to address women's health and well-being needs. 2. Identify optimal service settings for trauma discussions. 3. Outline training needs for maternity care providers. Target population: Women in the perinatal period with previous trauma.
Stakeholder involvement	Engage relevant stakeholders in guideline development.	Stakeholders, including experts by experience, healthcare professionals, and voluntary sector representatives, were actively involved through workshops, interviews, and public consultation. The Research Collective provided iterative feedback on the framework.
Rigour of development	Use systematic methods to collect and synthesise evidence, formulate recommendations, and plan updates.	The framework was informed by: 1. A systematic review and qualitative synthesis (21). 2. EMPATHY study interviews (37). 3. Key documents [e.g., (17, 18)]. 4. Insights from the Research Collective. Recommendations were evidence-based and balanced potential benefits and risks.
Clarity of presentation	Ensure recommendations are specific, unambiguous, and clearly presented.	The framework was assessed for clarity by the Research Collective and through public consultation. Recommendations were refined to ensure they were specific, sensitive, and accessible. Language was adjusted to reflect diverse preferences (e.g., using “difficult experiences” alongside “trauma”).
Applicability	Identify barriers and facilitators to implementation and strategies for uptake.	A public consultation gathered feedback on the framework's practicality and relevance. Barriers (e.g., resource constraints) and facilitators (e.g., staff training) were identified. Recommendations were tailored to local needs and included strategies for implementation and evaluation.
Editorial independence	Ensure recommendations are free from bias or competing interests.	The framework's content was not influenced by the study funders (National Institute for Health Research and Wellbeing of Women). No members of the Research Collective had competing interests. Recommendations were developed independently and transparently.

### Consultation on the framework

In March 2023, the Research Collective participated in a workshop to review the draft framework and provide feedback via Google Forms, a secure and user-friendly platform. Eleven members evaluated the framework, assessing each recommendation for clarity, sensitivity, importance, and value. Participants also provided free-text comments on the feasibility and potential harms of the recommendations. Feedback was overwhelmingly positive, with minor revisions suggested to improve clarity and inclusivity.

Key changes based on stakeholder feedback included:

- Replacing the term “midwives” with “maternity care providers” to reflect the diverse range of professionals involved in trauma discussions.
- Adding a recommendation for an additional antenatal appointment focused on women's well-being, to address concerns about limited time, partner presence, and the lack of an established trusting relationship at booking appointments.
- Clarifying the language of several recommendations to ensure they were accessible and unambiguous.
- Resolving minor technical issues in the feedback form, such as a missing comment box.

The draft framework was refined through a public consultation process to gather feedback from a wider audience, including healthcare professionals, voluntary sector experts, and women with lived experience of trauma. Recruitment for the consultation commenced on May 25, 2023, and concluded on September 10, 2023. The consultation was conducted online using a survey format, which included questions about the clarity, relevance, and feasibility of the framework's principles and recommendations. Participants were also invited to provide free-text comments and suggestions for improvement. The consultation was promoted through professional networks, social media, and voluntary sector organisations.

In March 2024, the Research Collective reconvened for a final workshop to review and comment on the framework, which had been updated to incorporate feedback from the public consultation.

### Data analysis

Consultation data were analysed using descriptive content analysis, following the three-phase approach outlined by Vaismoradi, Turunen and Bondas (22).

1. Preparation phase: The data were read and re-read to enable the researcher to become familiar with the content.
2. Organising phase: Responses were grouped into preliminary categories (e.g., “valuable,” “essential,” “unfeasible”) which were then reviewed for consistency and refined collaboratively
3. Reporting phase: Findings were presented narratively and supported with illustrative quotations.

JC led the analysis of the consultation data. Analysis of consultation responses considered the possibility that participants might provide polite or supportive initial comments before offering critique; coding captured both supportive and critical perspectives. Emerging interpretations were discussed and refined collaboratively through multiple meetings with the author team.

## Findings

### Participants

The public consultation received 52 responses, including ten interview participants (two of whom were former Research Collective members), 28 individuals approached based on their expertise or interest, and 17 recruited via channels such as Twitter (now X) and conference presentations. While demographic information was not explicitly collected, based on participant familiarity and shared details, 49 respondents identified as female and three as male. Most participants were based in the UK, with additional representation from Cameroon (*n* = 1), the Netherlands (*n* = 1), and Japan (*n* = 1). Only one participant declined to be acknowledged in the published guidance.

Participants represented a range of professional backgrounds, including:

- Voluntary sector representatives from organisations such as the Birth Trauma Association (the only UK charity solely supporting those affected by traumatic birth), For Baby's Sake (supporting expectant parents experiencing domestic abuse), Birth Companions (addressing inequalities during pregnancy and early motherhood), and Birthrights (advocating for human rights in childbirth).
- Healthcare professionals, including obstetricians, midwives, and health visitors, many with expertise in supporting women with histories of abuse.
- Specialists in maternal mental health and safeguarding, including those working in Mother and Baby Unit settings.
- Diverse professionals such as commissioners, social workers, national advocates, clinical psychologists, childbirth educators, and compassionate inquiry practitioners.
- Researchers focused on maternity care for survivors of sexual violence and abuse.
- Midwifery educators.
- Trauma survivors, some of whom also held academic or voluntary sector roles or supported local Maternity Voices Partnerships.

### Content of the evidence-based framework

The final framework can be found in Appendix 1.

The framework includes a preamble emphasising the importance of collaborative development with stakeholders, including experts by experience, maternal mental health services, voluntary sector organisations, and maternity care providers. It underscores the need to prioritise women's choice, control, and agency throughout the process.

The framework is structured around six core principles:

1. Whole system approach: Routine trauma discussions should be integrated into maternity care as part of a broader system-wide transformation, supported by policy changes, training, and resource allocation. Policies should specify who will conduct discussions, when and where they will take place, and referral pathways. Resources should also support ongoing staff supervision and reflective practice.
2. Promote trauma awareness and access to support: Women should be informed about the potential impact of trauma on their well-being and offered access to support services. Multiple “light-touch” opportunities should be provided for women to discuss past experiences or mental health concerns. Where feasible, maternity services should provide an additional antenatal appointment focused specifically on social, emotional, and psychological wellbeing, giving women a private space to disclose previous trauma if desired. Women should also have access to independent support resources that do not require disclosure to healthcare providers.
3. Build trust and relationships: Trauma discussions must be conducted sensitively, with a focus on building trust and maintaining confidentiality. Discussions should allow sufficient time, be conducted in private, and, where possible, involve a known care provider. Women should be able to decline to answer questions and be informed of the limits of confidentiality. Documentation should respect women's wishes while adhering to safeguarding requirements.
4. Staff training and support: Healthcare providers require adequate training and ongoing support to conduct trauma discussions effectively and manage the emotional impact of disclosures, recognising that some staff will have personally suffered traumatic experiences. Training should be developed in partnership with experts by experience and specialist voluntary sector organisations, covering counselling skills, recognition of trauma effects, and local referral pathways. Staff should have access to ongoing reflective supervision or confidential counselling to support their well-being.
5. Locally tailored pathways: Trauma discussions should be adapted to local contexts, considering available resources and the specific needs of diverse populations. Services should address cultural, linguistic, and accessibility barriers and provide both local and national support options to ensure equitable care for all women.
6. Ongoing evaluation and improvement: Services should systematically evaluate the implementation and impact of routine trauma discussions and use these insights to refine trauma pathways. This includes monitoring staff training, proportion of women asked about previous trauma, referrals made, and feedback from women and staff, with attention to potential unintended consequences such as re-traumatisation or impacts on staff wellbeing.

The EMPATHY framework offers maternity services concrete guidance on how to routinely discuss previous trauma in the perinatal period. Its recommendations emphasise both organisational change and individualised care, ensuring discussions are sensitive, safe, and supportive. The underpinning evidence base and rationale for each recommendation are provided in Table 3.

### Findings from the public consultation

Full stakeholder feedback is provided in Supplementary Table 1. Of the 22 recommendations presented, 11 remained substantially unchanged, with minor adjustments for clarity. The remaining 11 were revised based on feedback, and one new recommendation was added: maternity services should develop a comprehensive written policy for routine trauma discussions, including provisions for implementation, communication, staff training, supervision, evaluation, and review.

The following section presents a summary of participants’ responses to open-ended questions about the framework, offering insights into their perceived value, feasibility, acceptability, potential impact on disadvantaged groups, and risk of harm. Each subsection includes illustrative quotes, with consultation respondents identified as R1, R2, and so on. Respondents have been identified by category (e.g., woman with lived experience, maternity care provider, maternity educator, voluntary sector expert) to provide context while maintaining confidentiality. Some respondents have overlapping roles and experiences, and may be represented in more than one category.

### Value of the framework

The majority of respondents regarded the framework as highly valuable for women who have experienced trauma. Participants described it as “*absolutely invaluable*” (R9, voluntary sector expert), with one noting, “*there is much that is very important and valuable in these guidelines*” (R8, maternity care provider) and another stating, “*I feel grateful to read these very well thought through and trauma-sensitive directions to talk with our clients about difficult experiences*” (R25, maternity care provider). One participant highlighted the transformative potential of the framework, suggesting that its implementation “*would lead to a dramatic shift in perinatal experiences and significantly reduce retraumatisation*” (R41, woman with lived experience).

The framework was seen as addressing a critical gap in current practice. While awareness of trauma-informed care is growing, respondents noted that “*there is much less available about what this means or looks like in practice*” (R9, voluntary sector expert). The inclusion of clear recommendations for training maternity care providers was particularly well-received. One participant even expressed interest in piloting the framework within their NHS trust, underscoring its practical relevance.

Although the framework was generally well received, some respondents identified areas for improvement. Suggestions included expanding its scope to address commissioning services and aligning it with existing safeguarding and domestic abuse guidance and training. Participants also stressed the importance of sensitive implementation and the establishment of robust support pathways, both of which have been addressed in the final version of the framework. While several respondents recommended extending the framework to include co-parents or partners affected by trauma, this falls outside the scope of the EMPATHY study.

The challenge of finding appropriate language to discuss trauma was another recurring theme. As one participant noted, “*not everyone will identify as a trauma survivor,*” even if they exhibit symptoms of post-traumatic stress disorder (R41, woman with lived experience). A respondent with expertise in sexual violence and maternity care described the framework as “*excellent*” (R36, woman with lived experience) but advocated for a stronger survivor voice in its implementation. They argued:

“I know this might seem unrealistic in a currently under-funded and over-stretched system, but survivors need to be instrumental in bringing about change—otherwise, it is not a trauma-informed approach” (R36).

### Feasibility of implementation

Respondents expressed mixed views on the feasibility of implementing the framework. Some believed it could be seamlessly integrated into existing practices, particularly given its alignment with mental health and emotional well-being assessments. However, others highlighted significant challenges, including resource constraints and the overwhelming demands on maternity services. One participant captured this sentiment succinctly: “*The NHS is tired, very very noisy with* “*change*” *initiatives and nothing really changing*” (R28, maternity care provider).

Despite these challenges, many respondents emphasised the importance of the framework, arguing that improving care for women who have experienced trauma is essential. As one participant stated plainly, “*If they aren’t* [achievable]*, something has to change*” (R31, woman with lived experience). Others acknowledged the inherent difficulties in changing practice, noting that “*there will never be a [right] time*” (R9, voluntary sector expert) and that partial implementation could still yield significant benefits*:* “*If even half the guidelines were implemented, that would make a huge difference*” (R41, woman with lived experience). Additionally, several respondents stressed the importance of continuity of carer, with one describing it as “*paramount*” (R52, maternity care provider) to enabling women to feel safe when disclosing previous trauma.

To enhance feasibility, participants suggested aligning trauma discussions with established workstreams on domestic abuse, safeguarding, and mental health. These areas already have specialist maternity care teams, guidelines, and a presence in mandatory training, making them a natural fit for integration. Strong leadership and the appointment of implementation champions were also seen as critical, with one participant proposing that a funded coordinator role could facilitate successful implementation (R50, voluntary sector expert).

### Acceptability

Participants generally agreed that women would find the framework acceptable if the rationale for trauma discussions was clearly communicated and handled with sensitivity. Even for those without personal trauma histories, such discussions were seen as an opportunity to “*help women share all manner of concerns*” (R49, maternity care provider), raise awareness, and reduce stigma. Respondents shared examples of women responding positively to trauma discussions, often expressing gratitude and understanding, even if they had not experienced trauma themselves.

Drawing parallels with routine domestic abuse enquiries, participants noted that trauma discussions are generally well-received. As one respondent observed, “*Women are very supportive if they think it will help other women*” (R32, maternity care provider). This suggests that, when framed appropriately, trauma discussions can foster a sense of solidarity and collective benefit.

Guideline respondents highlighted challenges in addressing trauma during booking appointments. One maternity care provider explained, “*we ask lots of questions at booking that relate to trauma but have not built up a trusting relationship at that point*” (R6), while another noted that “*the booking appointment…may not be the place as there may not be sufficient time to respond adequately*” (R21). Concerns about partner presence were also raised, with one respondent observing that “*Some women can still find it difficult to talk when their partner is in another room… I don't feel that within this time a relationship can be established and a women would want to disclose. Time is something that will need to be offered*” (R52, maternity care provider). In response, the framework was revised to recommend an additional antenatal appointment focused on women's well-being. This protected space allows women to disclose trauma when they feel ready and ensures adequate time for sensitive, meaningful discussion.

### Inequality and disadvantage

Most respondents believed the framework would particularly benefit women facing inequality and disadvantage, highlighting the complex interplay between trauma, inequality, and lack of support. One participant explained:

“Most definitely [the guidance would benefit women facing inequality and disadvantage]—as they have often suffered significant trauma, are more susceptible to traumas that arise with multiple disadvantage, and these could impact their current experiences of pregnancy, birth, and mothering. They may also have less knowledge or access to places where they can find support” (R40, voluntary sector expert).

The framework was seen as having the potential to improve care for vulnerable groups, including women seeking asylum, individuals from ethnic minorities, and those facing socio-economic challenges. One participant suggested that the approach outlined in the framework “*could be the most impactful way to challenge health inequalities and reach those people who do not have trust in the system*” (R9, voluntary sector expert).

However, some respondents raised concerns about barriers to disclosure within certain ethnic and socio-economic groups. As one participant noted, “*They are the ones least likely to disclose because of fears of consequences*” (R3, maternity care provider). Addressing language barriers, ensuring cultural safety, and maintaining ongoing anti-racist efforts were identified as essential to make the framework inclusive and effective for all. Additionally, several respondents recommended using inclusive language to acknowledge individuals who are biologically female but do not identify as women.

### Potential for harm

Most participants believed the framework itself was unlikely to cause harm, with comments such as “*no more so than current fragmented care*” (R19, maternity care provider), “*far less than the harm caused when we don’t know about previous trauma*” (R37, maternity care provider), and “*more harm comes from women suffering guilt and blame for experiences that were not their fault*” (R5, woman with lived experience).

However, participants expressed significant concern that inadequate implementation could undermine the framework's effectiveness. One participant warned*,* “*Of course there are harms from disclosures if they are not managed well or if there is not sufficient time/corners are cut*” (R9, voluntary sector expert). Others feared the guidance could become “*a tick-box exercise*” (R38, voluntary sector expert) or “*another document uploaded in a cloud that nobody looks at*” (R42, voluntary sector expert), potentially raising unrealistic expectations for both women and care providers.

Insufficient training was identified as a key risk, potentially leading to insensitive discussions or coercion, which could worsen women's experiences and deter future disclosures. Participants also highlighted the potential for inappropriate handling or recording of trauma disclosures, which might stigmatise women. Additionally, there were concerns about burdening maternity care providers with additional responsibilities without adequate resources or support, leading to low uptake of the guidelines. Respondents stressed the importance of providing emotional support for staff to manage the challenges associated with trauma discussions effectively, with one eloquently summing up the pressures on maternity staff and the imperative of providing support to maintain a healthy workforce:

‘The impact of the work they do, their own lived experience, the stretched systems they work in, the responsibilities they hold and the extreme emotions they are working with from one moment to the next—joy, fear, sadness, grief…….if we are going to develop, grow and sustain a healthy maternity workforce, this is essential.’ (R49, maternity care provider)

## Discussion

In the UK and internationally, trauma discussions in maternity care have traditionally relied on questionnaire-based methods, where service users are asked to disclose specific past experiences, such as childhood sexual abuse or domestic violence (23). In contrast, the EMPATHY framework represents a paradigmatic shift towards a holistic, emotionally-centred approach that prioritises trust, safety, and empowerment. Rather than relying on tools and checklists, it seeks to create a supportive environment in which women feel heard, respected, and in control of their care.

The framework was developed through a systematic review, qualitative synthesis, and stakeholder interviews. It defines the optimal conditions for trauma discussions and outlines the training required to support maternity staff. The study was guided by a critical participatory action research (CPAR) methodology, underpinned by critical social theory, to examine power dynamics and structural injustices. CPAR actively engages affected communities in the co-production of knowledge and aims to create meaningful societal change (24).

To facilitate this approach, a Research Collective was established, bringing together individuals with diverse forms of expertise, including lived experience, voluntary sector practitioners, and maternity care professionals. Grounded in critical social theory, the EMPATHY framework explicitly addresses the needs of underserved populations, including women facing language barriers, immigration-related vulnerabilities, or cultural obstacles to disclosure.

An intersectional lens further informed the framework's development, recognising that experiences of trauma and barriers to care are shaped by the interplay of multiple social identities, including race, class, immigration status, disability, and linguistic exclusion (25, 26). By acknowledging these intersecting forms of oppression, the framework seeks to promote equitable, culturally safe care that does not rely on disclosure as a prerequisite for support. A key innovation is its recommendation that all women—regardless of whether they disclose trauma—should be offered access to relevant information and support. This inclusive approach seeks to avoid placing the burden of disclosure on women, while ensuring their needs are still met (21).

The EMPATHY framework addresses a critical gap in existing policy guidance, which often centres on identifying and supporting women in current abusive situations, with limited consideration of past trauma (27, 28). Although the NHS England guide to trauma-informed perinatal care calls for “*early and respectful trauma screening and assessment for all*” (18), p. 34), it provides little direction on implementation. The EMPATHY framework contributes a structured yet flexible model, grounded in evidence and shaped by stakeholder input.

By prioritising cultural safety, inclusivity, and staff well-being, the framework provides a comprehensive resource to support maternity care providers in delivering compassionate, trauma-informed care. Its implementation has the potential to transform perinatal experiences, fostering positive emotional outcomes for women and their families. By creating a safe space for open dialogue, the framework is designed to empower women to share their histories on their own terms, reducing feelings of isolation and stigma. This approach therefore has the potential to not only enhance women's emotional well-being but also strengthens the therapeutic relationship between care providers and families, laying the foundation for positive perinatal experiences (29).

However, poor implementation of the framework carries significant risks. Several participants highlighted the potential for harm if services introduce trauma discussions without ensuring that appropriate referral pathways and support systems are in place. Inadequate training, limited follow-up options, or poorly managed disclosures may re-traumatise women or leave them without the support they need. Therefore, the framework should not be implemented in settings where effective referral pathways and support infrastructures are lacking. Without these, the well-intentioned use of trauma discussions may unintentionally exacerbate distress, undermine trust, and cause further harm. This underscores the critical importance of a whole-system approach that includes staff training, supervision, and access to specialist support as prerequisites for safe and ethical implementation.

As the framework was developed within the configuration of UK maternity services, some elements may require adaptation in health systems with different funding models, workforce structures or service pathways. However, the principles underpinning safe and sensitive trauma discussions may still have relevance internationally.

### Strengths and limitations

The EMPATHY framework addresses a critical gap in the literature by providing practical, evidence-based recommendations for routine trauma discussions during the perinatal period. A key strength lies in its development through a critical participatory action research approach, which ensured the active involvement of diverse stakeholders, including experts by experience, healthcare professionals, and voluntary sector representatives. Perspectives from over 1,600 women and 250 healthcare professionals were integrated through a systematic review, qualitative synthesis, interviews, and public consultation, enhancing the framework's validity and applicability.

Methodologically, the study is grounded in robust empirical evidence, combining findings from a systematic review and qualitative interviews. It is the first to integrate the perspectives of both women and maternity care professionals on routine trauma discussions, offering a comprehensive understanding of the challenges and opportunities involved. Rigorous search strategies and measures to minimise bias, such as positionality and reflexivity, further strengthen the reliability of the findings.

Finally, the framework goes beyond identifying issues to propose practical solutions, demonstrating a commitment to translating research into actionable policy and practice. These strengths collectively enhance the study's credibility and potential to advance trauma-informed care in perinatal settings.

Despite its contributions, the study has several limitations. Challenges in recruiting women with limited English proficiency may affect the broader applicability of the findings. Additionally, the lack of data on participants’ personal trauma histories raises the possibility that certain types of trauma were under- or overrepresented. Although efforts were made to encourage open discussion in Research Collective workshops, some members of the Collective may have felt inhibited in sharing their views, particularly in the presence of healthcare professionals.

## Implications for policy, practice, and research

### Implications for policy

The EMPATHY framework represents a critical, evidence-based resource for integrating routine trauma discussions into UK maternity care. To support its effective implementation, it should be embedded within national maternity guidance and backed by dedicated, ring fenced funding. This funding must extend beyond initial training to include delivery, ongoing supervision, and system-level coordination, particularly in light of persistent understaffing and resource constraints that threaten implementation fidelity.

Strategic investment in the framework has the potential to generate long-term savings by facilitating earlier access to mental health services and mitigating the intergenerational transmission of trauma. Equally, policies must prioritise comprehensive support structures for staff, including access to independent psychological support and clinical supervision. These supports are essential for preventing burnout and vicarious trauma and for sustaining trauma-informed care over time.

### Implications for practice

The EMPATHY framework offers clear, actionable guidance for embedding trauma discussions within maternity services. It advocates for a whole-systems approach, ensuring healthcare providers are equipped with the necessary skills, time, and confidence to approach these conversations sensitively and effectively. Central to the framework is a commitment to building trust and upholding women's autonomy and informed choice.

A key innovation is the recommendation for a dedicated antenatal appointment focused on mental health and emotional well-being, scheduled shortly after the first maternity care appointment. This allows time for trust-building, enables women to prepare for the conversation, and creates an opportunity to provide independent access to support. By demonstrating parity between physical and mental health, this appointment could address long-standing limitations in current practice and facilitate safer, more meaningful trauma discussions.

It is important to note that the framework has not yet been implemented. Several practical challenges identified in the study—including limited appointment time, variable continuity of care, insufficient supervision and referral pathways, and the need for appropriate training—may affect how the framework can be operationalised. Without adequate infrastructure, routine trauma discussions risk causing harm, potentially retraumatising women or exposing staff to ethical and emotional challenges for which they are unprepared.

Trauma-informed care must not become a symbolic gesture or a box-ticking exercise; successful implementation requires the ethical and practical readiness of the entire maternity care system. Reflecting on these implementation challenges in practice highlights the need for careful planning, resource allocation, and ongoing evaluation to ensure the framework achieves its intended impact.

### Implications for research

The development of the EMPATHY framework highlights several critical areas for further research. First, there is an urgent need to co-design culturally safe, context-specific tools for initiating trauma discussions in the UK. Existing tools, such as the Adverse Childhood Experiences (ACE) questionnaire, have been found to be inappropriate or potentially harmful when used in maternity settings. Research should prioritise collaborative development of resources that centre women's lived experiences and uphold trauma-informed principles.

Second, future research should focus on producing and evaluating national implementation materials. These include policies, training curricula, and women-centred information resources that are co-developed with stakeholders from practice, voluntary organisations, and communities with lived experience. Additionally, the prevalence and impact of trauma among maternity staff must be examined, to inform organisational strategies that support staff well-being and improve workforce retention.

Finally, a robust framework for monitoring and evaluation is crucial to ensure that the EMPATHY framework does not inadvertently cause harm and continues to meet the needs of diverse populations. Future research should focus on tracking and improving implementation over time. Key areas for investigation include developing clear evaluation metrics to assess clinical outcomes, practitioner adherence, and the quality of trauma discussions, as well as considering patient-reported outcomes such as satisfaction with care, sense of safety, and perceived support. Additionally, staff experience, including emotional impact, confidence, and training effectiveness, should be examined, alongside feedback mechanisms that enable continuous input from both healthcare providers and women receiving care. Equity monitoring is also necessary to assess how well the framework serves minoritised and underserved groups, using disaggregated data to address disparities. A structured, participatory approach to evaluation will be essential to ensure the framework remains responsive, ethically sound, and effective in real-world practice.

## Conclusion

The EMPATHY framework represents a significant step forward in trauma-informed perinatal care, addressing a critical gap in existing guidance and practice. By providing a structured yet flexible approach to routine trauma discussions, the framework offers practical solutions to improve care for women who have experienced trauma. Its emphasis on cultural safety, inclusivity, and staff well-being ensures its relevance across diverse populations and settings.

While the framework has the potential to transform perinatal experiences and reduce health inequalities, its successful implementation will require sustained investment in training, resources, and support for maternity care providers. Further research is needed to refine tools, develop national materials, and explore the impact of trauma on care providers.

Ultimately, the EMPATHY framework paves the way for a more empathetic and supportive approach to perinatal care, in which women feel empowered to seek support and maternity care providers are equipped to deliver compassionate, trauma-informed care.

## Data Availability

The raw data supporting the conclusions of this article will be made available by the authors, without undue reservation.

## Supplementary material

The Supplementary Material for this article can be found online at: https://www.frontiersin.org/articles/10.3389/fgwh.2025.1608174 /full#supplementary-material

## Appendix 1

Thank you to everyone who responded to the public consultation on the framework, including the following people:

Colette Eaton-Harris

Dr Jenny Patterson

Jill Miller

Kenny Gibson

Alexander Heazell

Masako Kageyama

Dr Nicole Stokoe

Kaat De Backer

Jenny Cunningham

Mary Clare Chapman

Lucy Maddox

Laura Seebohm

Didier Demassosso

Effie

Laura Latina

Emma Doherty

Zoe Darwin

Hannah de Klerk

Denise Marshall

Marie-Clare Balaam

Amanda Mansfield MBE

Jude Field

Jane Forman

Pippa Atkinson

Tamsin Bicknell-Morel

Memuna Sowe

Katie Morris

Jan Bostock

Maureen Treadwell

Allison Cummins

Louise Raven-Tiemele

Siofra Peeren

Aliya Porter

Jo Costello

Michael Lomax

Eleanor Laidlaw Brown

Shona Langley

Judith Rees

catherine randall

Alessia

Katherine Letley

Dr Justine Leach

Rebecca Brione

Joy Horner

Lucie McAuslan

Kate Greenstock

Rebecca Thomas

Judy Shakespeare

Jo Rhys-Davies

Bernie Pike

Nikki Wilson

Thank you to everyone else who supported the study, including as an interview participant.

**About the framework**

Over a quarter of pregnant women (∼150,000) each year in the UK have suffered trauma such as domestic abuse, adverse childhood experiences, or sexual assault (30). These experiences can have a lasting effect on mental and physical health, and impact pregnancy and parenting. Despite this prevalence and the potential consequences, discussing prior trauma is not standard practice in maternity care in the United Kingdom.

This framework offers a new model for trauma discussions, informed by meaningful engagement with trauma survivors and stakeholders. It aims to help maternity care providers raise the issue of previous trauma and provide appropriate follow-up. The framework was developed as part of the EMpowering Pregnant women Affected by Trauma HistorY (EMPATHY) study, a critical participatory action research study which was guided by a Research Collective of women with trauma histories, experts from the voluntary sector, and maternity care providers.

A systematic review and qualitative evidence synthesis was conducted which included 25 papers from five countries, representing the views of 1,602 women and 286 healthcare professionals and experts from the voluntary sector (21). Interviews were then undertaken with women with lived experience of trauma (*n* = 4), healthcare professionals (12), and voluntary sector experts (*n* = 7) (37).

The following sources informed the development of an evidence-based framework of guiding principles for the routine discussion of previous trauma in the perinatal period:

- Papers included in the systematic review and qualitative synthesis (21).
- Findings from the study interviews (37).
- The seminal conceptual document “SAMHSA's Concept of Trauma and Guidance for a Trauma-Informed Approach” (17).
- The “Good Practice Guide to Implementing Trauma-Informed Care in the Perinatal Period”, commissioned by NHS England and NHS Improvement (18).
- Insights from the Research Collective.

The framework was further developed through a rigorous public consultation with 52 responses from participants with diverse professional backgrounds, including:

- Voluntary sector representatives, including those linked with the Birth Trauma Association, For Baby's Sake, Birth Companions, and Birthrights.
- Obstetricians, midwives, and health visitors, many with expertise in supporting women with abuse histories.
- Specialists in maternal mental health and/or safeguarding, including in Mother and Baby Unit settings.
- Diverse professionals, including a commissioner, a social worker, a national advocate, and a clinical psychologist, a childbirth educator and a compassionate inquiry practitioner.
- Researchers dedicated to maternity care for survivors of sexual violence and abuse.
- Midwifery educators.
- Trauma survivors, some with academic or voluntary sector expertise or who supported their local Maternity Voices Partnership.

The framework contains 23 recommendations based on six core principles: 1. Routine trauma discussion should be introduced as part of a system-wide change; 2. Maternity care providers should let women know previous trauma can affect their well-being and help them access support; 3. Trauma conversations need to be carried out sensitively, to build trust and relationships; 4. Staff must be provided with adequate training and support; 5. Trauma discussions should be tailored to local needs and services; and 6. Services should systematically assess the implementation and impact of routine trauma discussions and seek to continuously improve trauma pathways based on these insights. By offering flexible principles, the framework supports providers in tailoring discussions to each woman's needs while reinforcing women's agency and autonomy.

The term “routine” indicates the need for trauma discussions to be part of care for every woman, avoiding the unconscious biases, stigmatisation, and missed opportunities for support that can result when clinicians only discuss trauma with women who they believe to be affected.

**Preamble to the framework**

Maternity care services should develop procedures for routine trauma discussions in close collaboration with a steering group comprising experts by experience, maternity care providers responsible for conducting trauma discussions, maternal mental health services, and local voluntary service organisations. The steering group should be intentionally inclusive and representative of various trauma types and member demographics.

To ensure that steering group members have adequate support, consideration should be given to recruiting experts by experience through voluntary service organisations. Participants in the steering group should receive compensation for their invaluable expertise and contributions. Feedback mechanisms, including anonymous options, should be implemented to foster open and inclusive communication within the group. The steering group should be meaningfully involved throughout the entire process of developing, implementing, and evaluating routine trauma discussions in maternity care.

The overarching principle of empowering women by promoting choice, control and agency over decisions relating to their care should be upheld at all times.

**Principle 1. Routine trauma discussion should be introduced as part of a system-wide change**

1. Maternity care services should develop a comprehensive written policy for routine trauma discussions, addressing the following key elements:
  - Who, how, when, and where discussions will take place.
  - Referral pathways.
  - Communication strategy to prepare women for trauma discussions, ensure they understand the purpose and benefits, and inform them of available support resources.
  - Strategies to ensure trauma discussions are culturally sensitive, equitable, and accessible. This includes addressing the needs of women with limited English proficiency or other communication needs and women who seek care later in pregnancy or have received limited maternity care.
  - Format, content, and delivery plan for staff training, including provisions for ongoing training to maintain competency and awareness.
  - Mechanisms for providing supervision and ongoing emotional support to staff involved in conducting trauma discussions.
  - Procedures for evaluating and monitoring the impact and acceptability of routine trauma discussions, incorporating feedback from both women and staff.
  - Identifying key individuals or teams responsible for implementing and overseeing the policy within maternity care services.
  - A regular review schedule for the policy, to ensure it is responsive to emerging research, evolving practices, and feedback from stakeholders.

**Principle 2. Maternity care providers should let women know previous trauma can affect their wellbeing, and help them access support**

2. Maternity care providers should make women aware that previous difficult or traumatic experiences can affect their current wellbeing and experience of pregnancy and parenting.
3. Discussions about difficult experiences should be combined with discussions about mental health, because many troubling thoughts, feelings, and behaviours are attributable to previous experiences.
4. Maternity care providers should give women multiple “light-touch” opportunities to talk about mental health concerns and previous difficult or traumatic experiences, because women may not feel comfortable disclosing or need support until later in the perinatal period.
5. Maternity care providers should only ask direct questions about difficult or traumatic previous experiences if there is a protocol and referral pathways in place and they have had training in how to ask and respond.
6. Women should be provided with information and support that they can access independently, without the need to disclose traumatic experiences to healthcare providers. Maternity care providers should address potential concerns about confidentiality, reassuring women that they cannot determine whether she has accessed online resources.
7. When women disclose previous difficult or traumatic experiences, maternity care providers should collaborate with them to develop a personalised plan of care for the perinatal period that prioritises choice, control, and individualised care. This plan could include:
  - Clarifying birth preferences or wishes.
  - Addressing potential triggers, with specialist psychological support if needed.
  - Facilitating continuity of carer where feasible.
  - Assisting in accessing mental health support if this would currently be, or might become, beneficial. In cases where women may not meet criteria for perinatal mental health services, exploring alternative support options such as third sector organisations or online resources is recommended.
  - Providing information about additional support services, such as peer support, parentcraft groups, third-sector, community, or online resources.
  - Offering information for women's partners on how to provide support during this time.

However, it is important to note that structured care plans may not be desired or beneficial for all women.

**Principle 3. Trauma discussions should be carried out sensitively, in a way that builds trust and relationships**

8. Women should be sensitively forewarned that the issue of previous trauma will be raised, providing them with the opportunity to prepare for the discussion and ensure they have adequate support in place. They should be informed that they can opt out of answering any questions about previous difficult experiences and told of the limits of confidentiality.
9. The issue of previous difficult or traumatic experiences should be raised when there is sufficient time for staff to listen and respond to disclosures, recognising that for women who do not feel listened to, these discussions can be re-traumatising. When care providers cannot adequately respond to a disclosure due to time constraints, they should acknowledge the disclosure and schedule a follow-up appointment where they will be able to talk in more depth. Service managers should ensure appointments include additional time for trauma discussions and facilitate autonomy in arranging follow-up or additional appointments.
10. An additional antenatal appointment specifically focused on addressing women's social, emotional, and psychological well-being, including the opportunity to disclose any previous traumatic events if desired, should be provided. This appointment should adhere to the following criteria:
  - Conducted in a private and undisturbed environment.
  - Without the presence of a partner, acknowledging that some women may not have disclosed their traumatic experiences to their partners or that partners may have been involved in the experiences. However, if a woman prefers to include her partner or a trusted support person in the discussion, a follow-up appointment should be offered.
  - Ensure there is a private space available and a dedicated staff member to provide support if a woman becomes upset during the conversation, allowing her the necessary time to gather herself.
  - Ideally conducted by a female care provider, recognising that some women may not feel comfortable disclosing previous trauma to male staff.

All maternity care settings should prioritise allocating resources to facilitate this additional appointment. If an additional appointment is currently not feasible, services should consider how the above points can be integrated within existing maternity care appointments.

11. Where possible, the issue of previous difficult or traumatic experiences should be raised by a maternity care provider who is known to the woman, as many women will not disclose trauma without a trusting relationship.
12. Maternity care providers should collaborate with women to ensure documentation of trauma disclosures is sensitive and acceptable (while adhering to safeguarding requirements), recognising and advising women that maternity records may inadvertently be viewed by others, including partners and family members. This approach aims to both prevent sharing of information without consent and reduce the potential for re-traumatisation by minimising the need for women to needlessly repeat their stories.
13. Maternity care providers should ask women's wishes about information sharing within the maternity team and with other services, and as far as possible follow these wishes.

**Principle 4. Staff should be given training and support to carry out routine trauma discussions**

14. Maternity care providers should undergo comprehensive training to sensitively conduct trauma discussions. This training must be collaboratively developed and delivered in partnership with experts by experience and specialist voluntary sector organisations, with due compensation for their invaluable expertise. Ongoing training, supervision, and support should be provided to staff to ensure sustained competence. The training curriculum should include the following key elements:
  - Understanding the potential effects of trauma on mental and physical health, behaviour, wellbeing, and parenting across diverse population groups.
  - Fundamental counselling skills, including active listening, employing open-ended questions, building confidence in asking about and responding to disclosures of difficult experiences, and sensitively concluding difficult conversations.
  - Recognising and sensitively supporting women who may have suffered trauma but choose not to disclose it.
  - Local care pathways available for women who have suffered trauma.
  - Appropriate documentation of trauma disclosures and safeguarding considerations.
  - An evaluation so the effectiveness and acceptability of the training can be monitored.

Facilitators of the training must be mindful that attendees may reflect on personal experiences, potentially eliciting painful memories, and should consider strategies to support them.

15. All staff working in maternity care, including support staff such as healthcare assistants and receptionists, should receive role-appropriate training in supporting women who may have suffered trauma.
16. Staff training on routine trauma discussion and trauma-informed care should begin in the undergraduate period.
17. Maternity care providers should be provided with regular (e.g., monthly) counselling, within paid working hours, to help them manage the emotional impact of discussions about trauma, including any personal memories these conversations may evoke. The counselling should be confidential and provided by a qualified professional who is independent of service management.

**Principle 5. Routine trauma discussions should be tailored to local needs and services**

18. Consideration should be given to overcoming cultural, systemic, and societal barriers to trauma discussions. These barriers include:
  - Shame, stigma, and silencing.
  - Expectations about gender.
  - Strong social taboos around discussing abuse, potentially leading to a lack of recognition of abusive experiences by women.
  - Lack of awareness of mental health issues.
  - Some languages lack specific vocabulary to describe mental health and may use terms that are stigmatising or derogatory (e.g., “crazy”).
  - Mistrust of institutions, which may stem from prior experiences with statutory services.
  - Fears that care providers will gossip or discuss their personal information without consent.
  - Cultural bias and racism from care providers.
  - Insecure immigration status, which can increase vulnerability to abuse and discourage disclosure of experiences.
  - Sexual orientation and gender identity.

To ensure these barriers are considered and to provide an inclusive approach, the development of pathways and the design and delivery of training should incorporate input from individuals with various cultural backgrounds and lived experiences.

19. Pathways should be designed with recognition of the specific challenges faced by women with limited English proficiency or other communication difficulties when disclosing trauma. These challenges may include:
  - Reluctance to disclose in the presence of an interpreter. It is essential to acknowledge and address potential barriers that interpreters might pose to open communication.
  - Fear that interpreters will breach confidentiality and disclose sensitive information to others in the community. Strategies should be implemented to build trust and ensure interpreter confidentiality.
  - Reluctance to disclose in the presence of partners, family, or friends who are acting as interpreters. It is crucial to discourage this practice, emphasising the importance of neutral and professional interpreters.
  - Limited literacy in their own language can mean translated materials are not helpful and make women feel ashamed. Services should strive to provide accessible information such as audio translations of questionnaires and information leaflets.
  - Difficulty understanding technical terms, written information, or subtle nuances even for women with good conversational English. Efforts should be made to communicate information in a clear, straightforward manner to ensure understanding across varying levels of English proficiency.
  - Services should also consider how they can meet the needs of women who have other communication needs, including hearing difficulties, learning disabilities, neurodivergence, or low literacy.
20. Routine trauma discussion pathways should be tailored to local resources and services. Women should also be informed of national support organisations to ensure a minimum level of support for all women, regardless of location. It is important to acknowledge that some women prefer anonymous support options, such as telephone-based or national rather than local services, due to concerns about confidentiality and social encounters with support providers. Additionally, poverty should be recognised as a barrier to accessing support.

**Principle 6. Services should systematically assess the implementation and impact of routine trauma discussions and seek to continuously improve trauma pathways based on these insights**

21. While respecting women's individual rights to confidentiality and their choices regarding documentation of trauma disclosures in medical records, efforts should be made to measure the uptake and impact of routine trauma discussions. Collected data could include:
  - Proportion of staff trained in conducting trauma discussions.
  - Proportion of women asked about previous trauma.
  - Basic sociodemographic information.
  - Number of women who disclosed trauma and types of traumas disclosed.
  - Changes in care resulting from trauma disclosures.
  - Uptake of referrals made.
  - Impact on related services such as referrals to mental health and addiction services.
  - Impact of routine trauma discussion on outcomes such as health, quality of life and experience of parenting.

In analysing the data, both the overall dataset and specific results relating to marginalised groups and individuals from different cultural backgrounds should be considered to ensure inclusivity and representation of diverse voices.

22. Feedback should be sought at a local level from women using maternity services and staff regarding routine discussion of previous trauma. The aim of this feedback is to establish whether it is acceptable and helpful, and to identify unintended consequences, such as the risk of re-traumatisation for women or negative impact on staff wellbeing. To encourage open communication and constructive criticism, feedback collection should be anonymous. Services should collaborate with voluntary service organisations to develop strategies to seek feedback from marginalised populations. Responses should be analysed both as a whole, and separately for marginalised groups and different cultural backgrounds, to ensure trauma discussions are equitable.
23. While upholding women's rights to confidentiality, maternity services should collaborate with each other to share findings and identify best practices. Findings should also be shared with the steering group, staff conducting trauma discussions, and local voluntary service organisations.
