# Supplementary material for: Empowering women through trauma-informed maternity care: the EMPATHY framework
Source: Front Glob Womens Health. 2025 Dec 4;6:1608174. doi: 10.3389/fgwh.2025.1608174 (PMC12715818; doi:10.3389/fgwh.2025.1608174)
Supplement: Supplementary file 1 [file Table1.docx]

**Table 1. Phases of the EMPATHY study**

| **Study phase** | **Participant numbers** |
| --- | --- |
| Systematic review and qualitative synthesis (Blinded for review., 2023) | 25 papers from 5 countries included, representing the views of 1602 women and 286 healthcare professionals and experts from the voluntary sector |
| Interviews  (Blinded for review, paper under review) | Women with trauma histories (n=4), healthcare professionals (n=12), and voluntary sector experts (n=7) |
| Public consultation on framework (described in this paper) | 52 respondents to the consultation |

**Table 2. Application of AGREE II quality domains in the development of the EMPATHY framework**

| **AGREE II domain** | **Description** | **How the domain was addressed in the EMPATHY framework** |
| --- | --- | --- |
| **Scope and purpose** | Clearly define the aim, health questions, and target population. | The framework aims to:  1. Provide guidance on sensitive and effective trauma discussions to address women’s health and well-being needs.  2. Identify optimal service settings for trauma discussions.  3. Outline training needs for maternity care providers.  Target population: Women in the perinatal period with previous trauma. |
| **Stakeholder involvement** | Engage relevant stakeholders in guideline development. | Stakeholders, including experts by experience, healthcare professionals, and voluntary sector representatives, were actively involved through workshops, interviews, and public consultation. The Research Collective provided iterative feedback on the framework. |
| **Rigour of development** | Use systematic methods to collect and synthesise evidence, formulate recommendations, and plan updates. | The framework was informed by:  1. A systematic review and qualitative synthesis (Cull et al., 2023).  2. EMPATHY study interviews.  3. Key documents (e.g., SAMHSA, 2014; Law et al., 2021).  4. Insights from the Research Collective. Recommendations were evidence-based and balanced potential benefits and risks. |
| **Clarity of presentation** | Ensure recommendations are specific, unambiguous, and clearly presented. | The framework was assessed for clarity by the Research Collective and through public consultation. Recommendations were refined to ensure they were specific, sensitive, and accessible. Language was adjusted to reflect diverse preferences (e.g., using “difficult experiences” alongside “trauma”). |
| **Applicability** | Identify barriers and facilitators to implementation and strategies for uptake. | A public consultation gathered feedback on the framework’s practicality and relevance. Barriers (e.g., resource constraints) and facilitators (e.g., staff training) were identified. Recommendations were tailored to local needs and included strategies for implementation and evaluation. |
| **Editorial independence** | Ensure recommendations are free from bias or competing interests. | The framework’s content was not influenced by the study funders (National Institute for Health Research and Wellbeing of Women). No members of the Research Collective had competing interests. Recommendations were developed independently and transparently. |

Table 3. Evidence base for the framework of guiding principles for routine trauma discussions in the perinatal period

| **Final recommendation and *rationale*** | **Underpinning evidence (study numbers as per  table 3.2)** | **Feedback from guideline consultation** |
| --- | --- | --- |
| **1**. Maternity care services should develop a comprehensive written policy for routine trauma discussions, addressing the following key elements:   - Who, how, when, and where discussions will take place. - Referral pathways. - Communication strategy to prepare women for trauma discussions, ensure they understand the purpose and benefits, and inform them of available support resources. - Strategies to ensure trauma discussions are culturally sensitive, equitable, and accessible. This includes addressing the needs of women with limited English proficiency or other communication needs and women who seek care later in pregnancy or have received limited maternity care. - Format, content, and delivery plan for staff training, including provisions for ongoing training to maintain competency and awareness. - Mechanisms for providing supervision and ongoing emotional support to staff involved in conducting trauma discussions. - Procedures for evaluating and monitoring the impact and acceptability of routine trauma discussions, incorporating feedback from both women and staff. - Identifying key individuals or teams responsible for implementing and overseeing the policy within maternity care services. - A regular review schedule for the policy, to ensure it is responsive to emerging research, evolving practices, and feedback from stakeholders.   *This recommendation aims to provide a structured framework for providing routine trauma discussions, ensuring consistency, clarity, and effectiveness in practice.* | This recommendation consolidates the previously described evidence. NHS England and NHS Improvement advocate integrating trauma-informed care into organisational culture, policies, and practices, with input from experts by experience (Law et al., 2021). SAMHSA (2014) further recommends incorporating a trauma focus into policies and procedures. | Not applicable - this recommendation was added after the consultation. |
| **2**. Maternity care providers should make women aware that previous difficult or traumatic experiences can affect their current wellbeing and experience of pregnancy and parenting.  *Enables appropriate support to be put in place and prepares women for potential challenges that may arise during this critical period.* | NHS England and NHS Improvement advocate for providing women with information on the impact of prior trauma in the perinatal period, along with available support (Law et al., 2021).  The EMPATHY study's systematic review and qualitative synthesis highlight the value of routine trauma discussions, as perceived by women and maternity care providers (1,2,3,4,5,6,7,8,10,12,13,14,15,16,17,18,19,20,21,22,23,24,25).  Participants in the EMPATHY study interviews believed that carefully navigating discussions around trauma and providing effective support post-disclosure could prepare women for unexpected distress in the perinatal period and facilitate healing and growth. They highlighted the potential of trauma discussions to break the cycle of intergenerational trauma and tailor care to individual needs, while emphasising the economic benefits of well-funded interventions. | Participants proposed that trauma discussions can offer *‘clarity, reassurance, and relief’* (R41) to survivors, while also normalising the effects of trauma on pregnancy, birth, and parenting experiences.  However, participants stressed the need for sensitivity to avoid inducing anxiety or stigma and highlighted the importance of universally initiating trauma discussions without singling out women based on assumptions to prevent inadvertent stigmatisation.  Furthermore, they advocated for a balanced approach that acknowledges the potential for healing during pregnancy, birth, and parenthood, while avoiding overemphasis on negative experiences that may disempower survivors. |
| **3**. Discussions about difficult experiences should be combined with discussions about mental health, because many troubling thoughts, feelings and behaviours are attributable to previous experiences.  *Integrating these discussions could help establish a connection for women between previous traumas and ongoing mental health challenges.* | SAMHSA (2014) and NHS England and NHS Improvement (Law et al., 2021) highlight the close relationship between trauma and mental health problems.  The EMPATHY study’s systematic review found that some women were not fully aware of the extent or impact of the trauma they had suffered (1,2,3,4,6,12,13,14,18,22). Additionally, some women viewed their previous experiences as irrelevant to their current pregnancy (3,6,12,13,16,18,20).  Most participants in the EMPATHY study interviews advocated for combining discussions about mental health and trauma. They believed that such integration could support women's healing and growth by acknowledging the ongoing impact of past experiences. | Participants stressed the significance of combining discussions about challenging past experiences with conversations on mental health, highlighting their interdependence. They suggested that such dialogues might mitigate shame or stigma for women struggling with mental health issues. It was proposed that broaching either topic could facilitate disclosure of the other, noting that discussions about low mood could serve as a gateway for discussing traumatic experiences, and vice versa (R39).  However, respondents cautioned against assuming a direct link between all challenging experiences and ongoing mental health issues. They stressed the need for sensitivity, highlighting that not all individuals facing mental health problems have suffered trauma, and that not all difficult experiences continue to affect well-being.  Some participants expressed concerns about the lack of sustained support available for trauma survivors and the inadequacy of mental health services in responding to trauma. Others highlighted the necessity of clearly delineating role boundaries for maternity care providers and the need for improved training in this area. |
| **4**. Maternity care providers should give women multiple ‘light-touch’ opportunities to talk about mental health concerns and previous difficult or traumatic experiences, because women may not feel comfortable disclosing or need support until later in the perinatal period.  *Providing multiple opportunities to discuss trauma gives women choice and control over when to discuss their experiences, addresses the challenge of trauma discussions being unexpected, and enables trust to be established with maternity care providers.* | The EMPATHY study's systematic review highlighted the significance of choice and control for women, indicating that a flexible approach to trauma discussions that accommodates women's preferences for when to talk about these experiences is consistent with their needs (1,10,12,14,18,19). Providing multiple opportunities for women to share their experiences can address the challenge that trauma discussions can be unexpected (2,6,13,14,16,20).  Further, the review found that women may fear judgment if they disclose their histories, and giving them the chance to talk about difficult experiences later in pregnancy, when trust is established, may mitigate this fear (1,2,3,12,13,14,17,18,19,20,22,24). Additionally, the perinatal period can be unexpectedly challenging, making issues seemingly insignificant early in pregnancy more relevant later (1,2,4,10,12,14,18,19,20,24).  EMPATHY study interview participants stressed the importance of a patient and gradual approach to trauma discussions, recognising that it may require multiple appointments for women to feel comfortable disclosing their experiences. They recommended that practitioners keep the possibility of future disclosures open, allowing survivors the necessary time and space to decide when and how to share their experiences. Additionally, participants suggested that some women might not be ready to seek support until after the birth of their baby. | Participants unanimously supported offering multiple opportunities for discussions on mental health and trauma, believing these discussions would help *‘make every contact count’* and reduce stigma surrounding trauma (R38). Empowering women to decide when to engage in these conversations, rather than restricting them to a single opportunity, was seen as essential. The idea of *‘leaving a door open or planting a seed’* emerged, allowing women to reflect on their experiences and share when ready (R52).  However, opinions varied on the term 'light-touch', with concerns about ambiguity and potential trivialisation of trauma. Participants stressed the need for clear language and tools for maternity care providers to facilitate these conversations effectively. They also highlighted the importance of ensuring that women perceive these 'light touches' as invitations to share their experiences rather than mere conversational points.  Many participants observed that offering multiple opportunities to discuss past trauma allows time to foster trust between care providers and women. Continuity of care was highlighted as important for trust-building, allowing providers to "check in" at each visit and detect subtle changes in the woman's well-being (R30). However, some participants suggested that a new care provider might be better positioned to encourage disclosure due to different interpersonal dynamics. |
| **5.** Maternity care providers should only ask direct questions about difficult or traumatic previous experiences if there is a protocol and referral pathways in place and they have had training in how to ask and respond.  *This recommendation aims to mitigate the risk of lasting harm through insensitive trauma discussions.* | Both SAMHSA (2014) and NHS England and NHS Improvement (Law et al., 2021) advise universal trauma screening. However, the evidence base does not indicate a clear ‘right way’ to raise the issue of previous trauma. The EMPATHY study systematic review found with very low confidence that women favour a broad, conversational approach to discussing trauma (1,2,9,12,13).  Many participants in the EMPATHY study interviews questioned the effectiveness of quantitative trauma and mental health screening tools, feeling that a general, open style of conversation is more likely to elicit disclosure. Interviewees further proposed that closed-ended questions may deter women from disclosing because of a fear of social services involvement. It was also felt that direct questions could cause women to confront past experiences in an unanticipated and harmful way.  The EMPATHY systematic review underscored the critical importance of allocating sufficient time and resources to support routine trauma discussions (2,3,4,5,7,8,14,15,16,17,19,20,21,22,23,24). This finding resonates with insights gained from the EMPATHY study interviews, in which participants stressed that the value of trauma discussions lies in improving care, cautioning against mere documentation without follow-through. The recommendation aligns with the NHS England and NHS Improvement guide to implementing trauma-informed perinatal care, which emphasises the need for clear referral pathways for support relating to prior trauma (Law et al., 2021). SAMHSA (2014) advocates for a written policy or protocol for a trauma-informed approach for organisations, discouraging reliance on training workshops or individual leaders.  Both SAMHSA (2014) and NHS England and NHS Improvement (Law et al., 2021) emphasise the importance of staff training in how to carry out trauma discussions. In the EMPATHY study systematic review, twelve studies highlighted the critical role of effective communication skills among maternity care providers in eliciting disclosure while minimising distress experienced by women (1,2,3,6,8,13,14,16,18,19,22,24). Furthermore, three studies included in the review found that if not handled sensitively, trauma discussions could affect future health care access and experiences (12,14,16). EMPATHY study interview participants argued that maternity care providers should receive training in sensitively conducting discussions about previous trauma. They highlighted the potential for harm to be caused by trauma discussions, through insensitivity, inadequate support for women, or overzealous safeguarding responses. | Views on directly asking about previous trauma varied. Many agreed that direct questions should only be asked when care providers have received training and there is a structured protocol with established referral pathways, with one describing the *‘potential for a woman's experience to be made worse if she is encouraged to share and then does not receive the care that she needs’* (R16). Some respondents felt that all care providers should be able to ask generic ‘screening’ questions, but detailed conversations about previous trauma should be handled by specially trained care providers.  Others believed that maternity care providers already have the skills to conduct sensitive trauma discussions. They highlighted that *‘the act of asking sensitively and hearing a disclosure can be incredibly powerful and healing in and of itself’* (R36), even in the absence of further support or referral. Concerns were raised regarding the absence of clear protocols in many areas, potentially hindering providers from initiating direct enquiries and consequently depriving women of essential support. Participants expressed concerns that care providers might use the lack of established referral pathways to avoid engaging in these conversations.  The recommendation is a pragmatic compromise: while acknowledging that the lack of established procedures in some areas may result in women not being asked about previous trauma, potentially hindering their access to support, it aims to mitigate the risk of lasting harm through insensitive trauma discussions. The recommendation underscores the imperative of implementing protocols and training to ensure that all women have an opportunity to discuss previous trauma and receive the support they need. Following the guideline consultation, the reference to a private setting was removed as this is covered in recommendation 9. |
| **6**. Women should be provided with information and support that they can access independently, without the need to disclose traumatic experiences to healthcare providers. Maternity care providers should address potential concerns about confidentiality, reassuring women that they cannot determine whether she has accessed online resources.  *Providing access to information and support resources that do not necessitate disclosure empowers women to seek assistance on their own terms, respecting their autonomy and privacy.* | Evidence from the systematic review indicates that many women choose not to disclose previous trauma due to various reasons, such as distrust of the person asking, fear of judgement, closed-ended questions, time constraints, and a desire for privacy (2,3,4,6,10,12,13,14,16,17,18,19,24). This underscores the importance of offering alternative avenues for accessing support to ensure that all women, regardless of their disclosure choices, can receive the assistance they may require.  EMPATHY study interview participants emphasised the necessity of providing women with independent access to support, enabling them to seek help privately if they choose not to disclose their trauma histories. This recommendation aligns with NHS England and NHS Improvement guidelines, which advocate for providing all women with information on how prior trauma may impact them in the perinatal period, along with available support options (Law et al., 2021).  Interview participants also highlighted the importance of adopting a universal precautions approach, wherein healthcare providers assume that all women may have experienced trauma and take steps to minimise the potential for re-traumatisation. This approach is endorsed by SAMHSA and NHS England and NHS Improvement guidance (SAMHSA, 2014; Law et al., 2021). | Feedback from the EMPATHY guideline consultation overwhelmingly supported the importance of providing information and support to women without requiring disclosure of their experiences. Participants stressed that this approach could alleviate the burden of *‘suffering in silence’* (R2) for women who feel unable to disclose and empower them to access support on their own terms. Recommendations included providing information in diverse formats and languages, co-designed with service users and specialist organisations, and disseminating it through various channels to ensure accessibility and visibility. |
| **7.** When women disclose previous difficult or traumatic experiences, maternity care providers should collaborate with them to develop a personalised plan of care for the perinatal period that prioritises choice, control, and individualised care. This plan could include:   - Clarifying birth preferences or wishes. - Addressing potential triggers, with specialist psychological support if needed. - Facilitating continuity of carer where feasible. - Assisting in accessing mental health support if this would currently be, or might become, beneficial. In cases where women may not meet criteria for perinatal mental health services, exploring alternative support options such as third sector organisations or online resources is recommended. - Providing information about additional support services, such as peer support, parentcraft groups, third-sector, community, or online resources. - Offering information for women's partners on how to provide support during this time.   However, it is important to note that structured care plans may not be desired or beneficial for all women.  *This recommendation aims to ensure women are empowered to navigate the perinatal period with agency and support.* | The recommendation is supported by findings from the EMPATHY systematic review, which highlighted the intense and challenging nature of the perinatal period, potentially triggering memories of past trauma (1,2,4,10,12,14,18,19,20,24). Additionally, the review revealed that some women who have experienced trauma desire therapeutic support during this time (1,3,6,10,14,22,24). Additionally, some women who have suffered trauma want relationship-based care (1,6,10,12,14,18). Moreover, the perinatal period holds potential for healing and growth (1,4,6,8,12,18,19).  Feedback from EMPATHY study interview participants supported the value of creating a care plan, including information for partners on how to offer support during this period. They noted that some, but not all, women may find value in groups such as parentcraft or peer support groups.  The recommendation aligns with guidance on trauma-informed perinatal care from NHS England and NHS Improvement (Law et al., 2021), which advises that all women should be supported to develop a personalised care and support plan. | Consensus emerged among respondents on the importance of creating a comprehensive perinatal care plan subsequent to trauma disclosures. Participants underlined the need for a collaborative approach that grants women autonomy, control, and agency, acknowledging that not all survivors might wish for or benefit from a structured care plan. Participants recommended offering information about additional support services such as peer support, third-sector, community, or online resources. Recognising that many women might not meet the criteria for perinatal mental health services post-trauma, the suggestion was made for services to explore ways to cater to these individuals' needs.  Participants advocated for using terms such as ‘birth preferences’ or ‘wishes’ instead of ‘plan’, extending the scope of plans beyond birth to encompass postnatal considerations and strategies for social support, and involving partners. Offering sample plans outlining potential triggers and practical adjustments was recommended, as well as providing specialist support for navigating complex discussions about triggers and management strategies. Ensuring these plans were respected and followed by care providers was felt to be crucial in preventing women from feeling *'violated and betrayed'* (R36)*.*  In response to the consultation feedback, the recommendation was refined to acknowledge that structured care plans may not be desired by all women, terminology was modified to ‘birth preferences or wishes’, support was included for women who do not meet the threshold for perinatal mental health services and guidance provided for helping partners support women. |
| **8.** Women should be sensitively forewarned that the issue of previous trauma will be raised, providing them with the opportunity to prepare for the discussion and ensure they have adequate support in place. They should be informed that they can opt out of answering any questions about previous difficult experiences and told of the limits of confidentiality.  *Sensitively forewarning women about forthcoming trauma discussions, providing them with the choice to participate, and clearly delineating confidentiality boundaries fosters agency and empowerment.* | The EMPATHY systematic review identified that routine trauma discussions can be perceived as invasive and unexpected by some women (2,6,13,14,16, 20), with choice and control being vital components of their experiences (1,10,12,14,18,19). Moreover, studies within the review highlighted that insensitive handling of trauma discussions may impact future healthcare interactions (12,14,16).  EMPATHY study interviewees emphasised the importance of forewarning women about forthcoming trauma discussions, allowing them to prepare and secure adequate support. While discussing confidentiality boundaries was felt to foster trust, concerns were raised that forewarning might induce anxiety, potentially leading to disengagement from care.  Guidance from NHS England and NHS Improvement and SAMHSA underscores the importance of effective communication, empowering women to make informed decisions about their care (Law et al., 2021; SAMHSA, 2014). | Participants emphasised the need to prepare women for trauma discussions, offer them the choice to decline discussing their experiences, and clearly define confidentiality boundaries. While transparency, choice, and control were highlighted as essential aspects of trauma-informed care, concerns were raised about the potential anxiety induced by pre-warning and its impact on appointment attendance. Others argued that an 'opt-out' approach might inhibit further discussions. Some participants instead suggested a gentle approach, introducing sensitive topics during routine discussions to foster a safe and comfortable environment where women feel empowered to engage when they are ready.  In response to consultation feedback, the recommendation was refined to explain the rationale for preparing women for trauma discussions and emphasise the importance of sensitivity in forewarning. |
| **9.** The issue of previous difficult or traumatic experiences should be raised when there is sufficient time for staff to listen and respond to disclosures, recognising that for women who do not feel listened to, these discussions can be re-traumatising. Where care providers cannot adequately respond to a disclosure due to time constraints, they should acknowledge the disclosure and schedule a follow-up appointment where they will be able to talk in more depth. Service managers should ensure appointments include additional time for trauma discussions and facilitate autonomy in arranging follow-up or additional appointments.  *This recommendation aims to prevent harm from trauma discussions where women do not feel listened to, and ensure adequate support is provided for staff to conduct sensitive and effective conversations about trauma.* | The EMPATHY study’s systematic review found with high confidence that support for routine trauma discussion is contingent on having adequate time for the discussion (2,3,4,5,7,8,14,15,16,17,19,20,21,22,23,24). Furthermore, studies included in the review found that mishandled trauma discussions could impact future health care access and experiences (12,14,16).    Multiple participants in the EMPATHY study interviews stressed that clinicians should broach the topic of previous difficult experiences only when they can allocate sufficient time to listen and respond to disclosures. They highlighted the unpredictable nature of trauma conversations, with some discussions requiring significant time. Participants emphasised the profound significance of trauma conversations for women, especially if they have never shared these experiences before, and stressed the importance of providing sufficient space for such discussions.  Guidance by NHS England and NHS Improvement (Law et al., 2021) states that services should facilitate disclosure by ensuring adequate time for women to discuss their needs. | Participants emphasised the need for staff to dedicate adequate time to listen and respond when women share previous difficult experiences. They highlighted that rushed or inattentive conversations could diminish the significance of these disclosures and erode trust in maternity care. One respondent expressed this, saying, *‘it's insulting if someone asks you questions about abuse then doesn't have time to listen’* (R5). However, concerns were raised that this recommendation might be misused by care providers to avoid challenging conversations. Respondents suggested that when a care provider’s workload prevents in-depth discussion, they should acknowledge the disclosure and schedule a follow-up appointment for a more thorough conversation. Participants stressed the role of management in ensuring staff have manageable workloads. The importance of continuity of carer was also highlighted, offering multiple opportunities for discussing trauma at more suitable times. One respondent proposed the idea of establishing a specialist midwife post or team to support women who have experienced trauma, expressing concerns about inadvertently re-traumatising women with well-meaning but under-resourced initiatives.  In response to feedback from the consultation, the guideline was revised to address time constraints for staff responding to disclosures and emphasise service managers' responsibility to allocate sufficient time for trauma discussions. |
| **10.** An additional antenatal appointment specifically focused on addressing women's social, emotional, and psychological well-being, including the opportunity to disclose any previous traumatic events if desired, should be provided. This appointment should adhere to the following criteria:   - Conducted in a private and undisturbed environment. - Without the presence of a partner, acknowledging that some women may not have disclosed their traumatic experiences to their partners or that partners may have been involved in the experiences. However, if a woman prefers to include her partner or a trusted support person in the discussion, a follow-up appointment should be offered. - Ensure there is a private space available and a dedicated staff member to provide support if a woman becomes upset during the conversation, allowing her the necessary time to gather herself. - Ideally conducted by a female care provider, recognising that some women may not feel comfortable disclosing previous trauma to male staff.   All maternity care settings should prioritise allocating resources to facilitate this additional appointment. If an additional appointment is currently not feasible, services should consider how the above points can be integrated within existing maternity care appointments.  *This approach aims to facilitate disclosure and prevent harm caused by insensitive trauma discussions.* | EMPATHY study participants identified challenges in discussing trauma during the booking appointment. They proposed that this appointment is already overcrowded and lacks sufficient time for meaningful conversations, mainly consisting of closed questions and information-giving by the maternity care provider, making it hard to transition to sensitive discussions. Interviewees proposed that the intrusive nature of questions asked during this appointment, including those about previous terminations, drug use, mental health, and social services involvement, can deter women from disclosing trauma. The EMPATHY study systematic review found that embedding trauma discussion in routine practice is challenging (4,5,7,15,17,21,23).  Interview participants also observed that the clinical environment of a booking appointment is not conducive to sensitive discussions, and they proposed that discussing trauma in front of partners, who may be unaware of the woman’s history, is not appropriate. Studies included in the EMPATHY systematic review recommended holding trauma discussions in private, comfortable, and welcoming surroundings (3,6,14,22) and highlighted partner presence as a barrier (7,21). NHS England and NHS Improvement (Law et al., 2021) proposed that services should consider the care environment's suitability for disclosure, including privacy and the presence of partners or family members. SAMHSA (2014) stressed the need for a safe, calm environment for trauma-informed care.  EMPATHY study participants favoured an additional antenatal appointment focusing on emotional health and trauma discussions. They believed this could create a protected space for meaningful conversations, proposing an unstructured, woman-led format similar to 'listening visits' for mental health support. Moreover, participants believed that an additional appointment could facilitate trust and relationship-building between the woman and the care provider. They proposed that women might be more inclined to disclose trauma during this second appointment, particularly if it involved the same care provider. Discussing trauma at the second appointment would allow women to be prepared for the conversation, avoiding overwhelming them during the initial appointment, which typically involves various medical procedures and enquiries.  Most EMPATHY study interview participants preferred female clinicians, citing personal comfort and trust as key factors. Some participants expressed directly that they would not disclose to a male clinician. SAMHSA (2014) advises that services provide gender-specific trauma screening, assessment, and treatment. NHS England and NHS Improvement (Law et al., 2021) state that services should offer the choice of female staff to enhance women's comfort and willingness to disclose trauma. | Respondents generally agreed that trauma discussions should take place in a private and undisturbed environment. Views on partner presence varied, recognising partners as potential sources of support or, in some cases, of abuse, and they may not be aware of a woman's trauma history. A pragmatic proposal emerged suggesting that women should be given the choice of having a support person present. One advocate for this approach remarked, ‘we wouldn’t want to recreate an experience of feeling alone, exposed and vulnerable’ (R48). Participants also recommended clarifying the nature of support for women who become distressed during these discussions. In response to feedback, the recommendation was amended to recognise women's preferences regarding partner involvement and outline support measures for those who become distressed during discussions.  The recommendation was revised subsequent to the consultation to propose an additional appointment specifically focused on women’s well-being, ideally conducted by a female care provider. Though not integrated into the guideline consultation, respondents indicated that these additions would be well-received. Guideline respondents identified challenges with addressing trauma during booking appointments, expressing concerns about the lack of a established trusting relationship at that stage, the risk of trauma discussions becoming perfunctory if included in this appointment, and the potential inadequacy of time to respond appropriately. Respondents noted that being cared for by exclusively female care providers is important to some women. |
| **11**. Where possible, the issue of previous difficult or traumatic experiences should be raised by a maternity care provider who is known to the woman, as many women will not disclose trauma without a trusting relationship.  *This approach aims to facilitate disclosure through creating trust.* | The systematic review and qualitative synthesis indicate with high confidence that for some women, relationships are a crucial factor in trauma disclosure (1,2,3,6,10,12,13,14,16,18,20,21,24). Six studies included in the review highlight that women who have suffered trauma prefer relationship-based care (1,6,10,12,14,18).  EMPATHY study interview participants overwhelmingly expressed that women are more likely to share past experiences within the context of a trusting relationship. They noted that multiple encounters may be necessary before women feel comfortable enough to disclose their histories. However, participants also acknowledged that clinicians can use kindness, compassion, and warmth to establish a psychologically safe environment, even without continuity of care.  NHS England and NHS Improvement (Law et al., 2021) advocate for continuity of carer to facilitate disclosure, recognise changes in mental health during the perinatal period, and reduce retraumatisation by minimising the need for repeated trauma disclosures to multiple practitioners. | Most participants supported the idea that maternity care providers known to the woman should initiate discussions about previous difficult or traumatic experiences. Respondents highlighted the importance of trust-building, particularly for women who have experienced domestic abuse, coercive control, or birth trauma. However, they also recognised that trust and rapport can be established even without prior meetings, with a respondent saying, *‘you can feel comfortable disclosing to someone on a first meeting if they appear approachable, interested, and trustworthy’* (R5). Some participants cautioned that continuity of care does not guarantee a warm and trusting relationship. Training care providers in rapid relationship building was suggested as a valuable skill that would enhance all aspects of care, not just trauma discussions. |
| **12.** Maternity care providers should collaborate with women to ensure documentation of trauma disclosures is sensitive and acceptable (while adhering to safeguarding requirements), recognising and advising women that maternity records may inadvertently be viewed by others, including partner and family. This approach aims to both prevent sharing of information without consent and reduce the potential for re-traumatisation by minimising the need for women to needlessly repeat their stories.  *This recommendation aims to foster trust between women and maternity care providers and to prevent harm resulting from insensitive handling of trauma disclosures.* | The systematic review and qualitative synthesis underscores the importance of choice and control for women (1,10,12,14,18,19). Additionally, three studies included in the review highlight that mishandling trauma discussions can impact future healthcare access and experiences (12,14,16).  Participants in the EMPATHY study stressed the critical role of effective and sensitive documentation in building trusting relationships with women and delivering quality care. Concerns were raised about inadvertent disclosure of trauma information to others, which could have significant repercussions for women. Some women prefer their experiences not to be documented, Interviewees suggested care providers work with women to document notes, ensuring they retain autonomy over their information as far as possible.  This recommendation aligns with guidance from NHS England and NHS Improvement and SAMHSA that women should be empowered to be involved and make decisions about their care (Law et al., 2021; SAMHSA, 2014). | Participants widely agreed on the importance of sensitive documentation, advocating for a collaborative approach where the woman and the care provider co-produce the record of their conversation. This ensures that the survivor's voice is heard and prevents the imposition of labels or interpretations that they may not agree with, such as categorising their experience as rape or domestic violence. However, concerns were raised about expecting emotionally distressed women to participate in the documentation process, suggesting that the onus should be on the provider to understand acceptable language and phrasing. Protective measures, such as keeping notes separate from routine records, were recommended to safeguard privacy and prevent access by controlling partners.  Documentation of trauma disclosures proved to be a complex and nuanced area. Several participants recommended care providers should document a deliberately vague statement acknowledging the experience of trauma, with a focus on the plan of care. Furthermore, there were suggestions that women should have the autonomy to decide whether their trauma history is documented at all. One participant pointed out, *‘often there is no reason for a disclosure of historical trauma to be documented, and it is critical that survivors feel ownership over their own narrative’* (R36). They expressed concerns that the absence of this autonomy could erode trust and deter survivors from seeking future services. However, it was noted that sensitive and comprehensive trauma documentation could potentially validate women's disclosures in future legal proceedings, highlighting a potential benefit of such documentation.  Participants stressed the importance of handling trauma information sensitively. One respondent vividly expressed feeling *‘exposed, ashamed, and angry that my joy in this pregnancy had been hijacked in such a cavalier way’* (R42) when a nurse in her second pregnancy referred to a rape she had disclosed in her first pregnancy in what she felt was a casual and dismissive manner. |
| **13.** Maternity care providers should ask women’s wishes about information sharing within the maternity team and with other services, and as far as possible follow these wishes.  *This recommendation is aimed at building trust between women and maternity care providers, improving communication among healthcare professionals, and mitigating harm resulting from insensitive handling of trauma disclosures.* | This recommendation is in line with findings from the systematic review and qualitative synthesis which emphasised the importance of choice and control to women (1,10,12,14,18,19).  In the EMPATHY study interviews, effective and consensual information sharing emerged as crucial in establishing trust and delivering quality care. This involves good communication between professionals, minimising the need for women to repeat their experiences unnecessarily, and ensuring clarity and agreement on the recipients of shared information.  Guidance from NHS England and NHS Improvement on trauma-informed perinatal care states that women should be empowered to make informed decisions about their care (Law et al., 2021).Similarly, SAMHSA guidance (2014) identifies empowerment as a fundamental principle of trauma-informed care. | The recommendation that care providers should ask women about their information sharing preferences received widespread support. Some participants believed it would help women appreciate the benefits of information sharing within the broader healthcare team, thereby contributing to optimal care provision. However, concerns were raised about safeguarding in cases where disclosure indicates potential risk of harm to the woman or her baby. Some participants suggested implementing a clear and upfront confidentiality statement before trauma discussions (addressed in recommendation 7).  One participant (R43) deemed the phrase ‘as far as possible’ too vague and open to interpretation, potentially leading healthcare providers to disregard a women’s wishes. Instead, they suggested aligning this recommendation with the legal requirements of safeguarding referrals: consent should always be sought from the individual before sharing their personal information with any other members of the maternity team and/or other services, unless disclosing such information would create a risk of further harm to them. |
| **14**. Maternity care providers should undergo comprehensive training to sensitively conduct trauma discussions. This training must be collaboratively developed and delivered in partnership with experts by experience and specialist voluntary sector organisations, with due compensation for their invaluable expertise. Ongoing training, supervision, and support should be provided to staff to ensure sustained competence. The training curriculum should include the following key elements:   - Understanding the potential effects of trauma on mental and physical health, behaviour, wellbeing, and parenting across diverse population groups. - Fundamental counselling skills, including active listening, employing open-ended questions, building confidence in asking about and responding to disclosures of difficult experiences, and sensitively concluding difficult conversations. - Recognising and sensitively supporting women who may have suffered trauma but choose not to disclose it. - Local care pathways available for women who have suffered trauma. - Appropriate documentation of trauma disclosures and safeguarding considerations. - An evaluation so the effectiveness and acceptability of the training can be monitored.   Facilitators of the training must be mindful that attendees may reflect on personal experiences, potentially eliciting painful memories, and should consider strategies to support them.  *This recommendation aims to equip care providers with the necessary skills to conduct sensitive and effective trauma discussions, thereby preventing harm caused by insensitive approaches.* | Twelve studies included in the EMPATHY study systematic review highlighted the critical role of effective communication skills among maternity care providers in eliciting disclosure while minimising women's distress (1,2,3,6,8,13,14,16,18,19,22,24). Additionally, three studies included in the review found that mishandled trauma discussions could impact future healthcare access and experiences (12,14,16).  Most EMPATHY study participants stressed the importance of interpersonal skills as a crucial training requirement. Guidance from SAMHSA and NHS England and NHS Improvement also emphasises the critical need for ongoing training on how to enquire and respond to disclosures (SAMHSA, 2014; Law et al., 2021).  EMPATHY study interview participants recommended training clinicians to recognise non-verbal indications of trauma or mental health struggles, an idea echoed by SAMHSA and NHS England and NHS Improvement (SAMHSA, 2014; Law et al., 2021). The NHS England and NHS Improvement guide recommends involving experts by experience in reviewing training, evaluating training to identify potential improvements, and considering the possibility that staff may be reminded of their own life experiences during training (Law et al., 2021). | The proposal that maternity care providers should receive training to conduct routine trauma discussions received widespread agreement, with one participant exclaiming, *‘yes, yes, a thousand times yes.’* Respondent R48 highlighted the critical role of training by sharing a personal experience where a lack of training led to problematic interactions. She stressed, *‘this is totally key I think - without training these questions can be awful.’* There was strong support for the involvement of survivors and specialist women-led organisations in developing and delivering this training, ensuring they are paid appropriately for their expertise. Several participants stressed the importance of ongoing training, supervision, and staff support to ensure the effectiveness of training.  In response to feedback from the consultation, the recommendation was expanded to involve survivors and specialist organisations, consider staff emotional wellbeing, specify that ongoing training should be provided, and evaluate effectiveness and acceptability of training. |
| **15**. All staff working in maternity care, including support staff such as healthcare assistants and receptionists, should receive role-appropriate training in supporting women who may have suffered trauma.  *Administrative and support staff play integral roles in the maternity care environment, directly influencing women’s care and experiences.* | The SAMHSA guideline for trauma-informed care advises that all staff, including those providing direct care, supervisors, receptionists, and cleaning and maintenance staff, receive basic training on the impact of trauma and trauma-informed approaches (2014). The NHS England and NHS Improvement trauma-informed care guide (Law et al., 2021) echoes this, proposing that both clinical staff, including sonographers, and non-clinical staff, including catering staff, can support women to feel safe in the care environment. EMPATHY study interviewees highlighted that any member of staff may notice signs of possible previous trauma, including administrative staff, and recommended that all staff be trained to recognise and communicate with clinicians about these observations. | The recommendation that all staff working in maternity care, including support staff, receive role-appropriate training in supporting women who may have suffered trauma gathered broad support. Training for support staff, including healthcare assistants and receptionists, was deemed essential for adopting a trauma-informed approach. Participant emphasised that these personnel may encounter signs of trauma or be the first to receive disclosures. Participants highlighted the importance of starting trauma-informed care from the initial contact with maternity services, as it could enhance women's experiences and engagement with care. |
| **16**. Staff training on routine trauma discussion and trauma-informed care should begin in the undergraduate period.  *Student maternity care providers play integral roles in the maternity care environment, directly influencing women’s care and experiences.* | EMPATHY interview participants advocated for training for maternity care providers to begin in the undergraduate period. This recommendation aligns with SAMSHA guidance (2014), which stipulates that all maternity services staff should undergo training in trauma impact and trauma-informed approaches. Additionally, NHS England and NHS Improvement guidance underscores the importance of ensuring that students and trainees feel adequately supported to deliver trauma-informed care (Law et al., 2021). | There was overwhelming consensus among participants that training on routine trauma discussions and trauma-informed care should commence during undergraduate education. Comments such as *‘yes this needs strongly embedded in the midwifery curriculum’* (R8), ‘AGREE AGREE AGREE’ (R47) and *‘100% it should be routine and standard’* (R11) showed strong agreement with this approach. Commencing training at this stage was viewed as a critical driver for systemic change and fostering a cultural shift towards trauma-informed care. Participants highlighted the importance of actively involving survivors and specialist women-led organisations in the development and delivery of this training, ensuring fair compensation for their valuable expertise. |
| **17**. Maternity care providers should be provided with regular (e.g., monthly) counselling, within paid working hours, to help them manage the emotional impact of discussions about trauma, including any personal memories these conversations may evoke. The counselling should be confidential and provided by a qualified professional who is independent of service management.  *This recommendation is aimed at supporting the emotional well-being of maternity care providers, acknowledging the challenging nature of trauma discussions and their potential to evoke personal memories. By offering regular counselling sessions within paid working hours, it ensures accessibility and normalises seeking psychological support. The requirement for confidentiality and independence from service management aims to create a safe space for staff to express themselves without fear of judgement or repercussion.* | This recommendation is underpinned by four essential elements: provision of regular emotional support to staff; support provided within paid working hours; independence from service management; and engagement with a qualified professional.  1. *Provision of regular emotional support*  The evidence consistently indicates provision of regular support is necessary for staff conducting trauma conversations. Five studies within the EMPATHY systematic review found that maternity care providers often find trauma disclosures distressing, impacting both their personal and professional lives (4,7,8,11,21). Clinical supervision was felt by participants to be vital for maternity care providers carrying out trauma discussions. EMPATHY study interviewees similarly talked of the burden of hearing upsetting stories of women’s difficult lives and suggested that awareness of this may make providers reluctant to engage in discussions about previous trauma. Discomfort could also cause providers to interrupt women, causing further distress to women and preventing future disclosures. SAMHSA (2014) propose that protecting staff well-being is a key element of a trauma-informed approach. NHS England and NHS Improvement (Law et al., 2021) emphasise the importance of regular supervision to prevent secondary traumatic stress caused by hearing trauma histories.  2. *Within Paid Working Hours*  Offering counselling sessions during paid working hours normalises seeking psychological support and ensures accessibility for all staff. Participants in the study carried out by Mollart, Newing and Foureur (2009) felt that clinical supervision is vital for care providers carrying out trauma discussions but noted that not all chose to access it. EMPATHY study interviewees pointed out that requiring an ‘opt-in’ to counselling will deter staff who fear this will portray them as weak or unable to cope. Interviewees further noted that some staff will not proactively seek support as they do not recognise the potential for burnout in their work. The majority of participants believed staff who are expected to carry out trauma discussions should be provided with regular reflexive supervision within working hours, making this a normal part of working life. This recommendation aligns with the NHS England and NHS Improvement guide to trauma-informed care, which states that protected time for supervision should be provided (Law et al., 2021).  3. *Independent of service management*  EMPATHY study interviewees expressed that many staff do not feel able to seek support from within the service due to concerns about confidentiality, judgement, and career implications. Further, interviewees expressed that where distress is being caused by personal memories or experiences, staff may be unwilling to discuss this with a colleague. SAMHSA (2014) also highlights the importance of supporting care providers affected by previous trauma.  4. *Professional support*  EMPATHY study interview participants noted that trauma disclosures may evoke significant distress, necessitating professional support. While the NHS England and NHS Improvement guide suggests utilisation of Professional Midwifery Advocates for staff support, I do not endorse this approach (Law et al., 2021). PMAs are not trained counsellors or therapists, lack independence from service management, and their dual role - supporting midwives while safeguarding the public - precludes guaranteeing confidentiality (NHS England, 2017). The guide also suggests peer support, but I have not included this in the recommendation due to insufficient evidence and an absence of an established model for trauma-affected staff (Law et al., 2021). | The recommendation for regular, independent counselling during paid working hours for maternity care providers received strong endorsement. Respondents unanimously agreed on the necessity of emotional support for staff, highlighting its value with responses like *‘this would be extremely valuable’* (R36), *‘this is vital and not a nice-to-do’* (R14) and *‘this is really important. I am so glad to see this’* (R37). They connected this support with improved care quality and staff retention. Participants highlighted the potential of routine trauma discussions to bring up personal memories and stressed the need to address this. They also recognised the possibility of vicarious trauma among staff, especially when supporting women through traumatic events. Participant R49 eloquently summed up the pressures on maternity staff and the imperative of providing support to maintain a healthy workforce:  *‘The impact of the work they do, their own lived experience, the stretched systems they work in, the responsibilities they hold and the extreme emotions they are working with from one moment to the next - joy, fear, sadness, grief.......if we are going to develop, grow and sustain a healthy maternity workforce, this is essential.’*  However, the feasibility of providing support within underfunded services was questioned by several participants, with comments including *'utopia indeed'* (R3) and *'while laudable, there are no resources to achieve this'* (R19). In contrast, others argued that staff support is an integral element of a trauma-informed approach and *‘cannot be seen as an added luxury’* (R9). Some suggested that informal peer support, supervision, or reflective practice would be suitable and cost-effective options. Group supervision was seen as helpful, but concerns were raised about potential dominance by strong personalities (R45). The wording of the recommendation was debated, with some suggesting that support should be optional rather than routine, and uncertainty expressed about the ideal frequency of support. |
| **18**. Consideration should be given to overcoming cultural, systemic, and societal barriers to trauma discussions. These barriers include:   - Shame, stigma, and silencing. - Expectations about gender. - Strong social taboos around discussing abuse, potentially leading to a lack of recognition of abusive experiences by women. - Lack of awareness of mental health issues. - Some languages lack specific vocabulary to describe mental health and may use terms that are stigmatising or derogatory (e.g., 'crazy'). - Mistrust of institutions, which may stem from prior experiences with statutory services. - Fears that care providers will gossip or discuss their personal information without consent. - Cultural bias and racism from care providers. - Insecure immigration status, which can increase vulnerability to abuse and discourage disclosure of experiences. - Sexual orientation and gender identity.   To ensure these barriers are considered and to provide an inclusive approach, the development of pathways and the design and delivery of training should incorporate input from individuals with various cultural backgrounds and lived experiences.  *This recommendation aims to facilitate trauma disclosure and mitigate harm caused by insensitive discussions, recognising the impact of cultural, systemic, and societal factors on individuals' ability to engage in open dialogue about trauma.* | EMPATHY study interview participants highlighted the significant role of cultural factors as a barrier to discussions about trauma. Participants noted that some languages lack the necessary vocabulary to articulate mental health issues, making it challenging to engage in sensitive conversations about them. Additionally, participants pointed out that the stigma surrounding mental health problems in specific cultures can impede open and constructive discussions on trauma. SAMHSA's concept of trauma and guidance for a trauma-informed approach (2014) underscores the importance of addressing cultural, historical, and gender considerations as fundamental principles of a trauma-informed approach. Building on this perspective, Law et al. (2021) stress the crucial role of cultural sensitivity in trauma-informed perinatal care. They recommend that care providers actively collaborate with local community groups to gain insights into cultural barriers to trauma-informed care. | Participants widely acknowledged the importance of considering cultural barriers when discussing past trauma. Comments like *‘this is crucial’* (R7), *‘this is so important’* (R41), and *‘excellent’* (R35) highlighted its significance. Participants particularly appreciated the inclusion of shame in these discussions. They suggested involving support workers and interpreters from diverse backgrounds in healthcare professional training. Recommendations were made to broaden the framework to include barriers faced by other groups, such as LGBTQIA+ individuals, women who have suffered FGM, and those with prior contact with statutory services. Participant R37 provided a valuable perspective, suggesting that terms like ‘racism from care providers’ and ‘stigma’ may be better framed as ‘cultural, systemic, and societal barriers’ to recognise the full range of obstacles. They also recommended adding ‘silencing’ to ‘shame and stigma’ to acknowledge that these are active processes that can keep survivors silent or silence them when they try to speak out.  As a result of feedback from the guideline consultation, the recommendation was amended to acknowledge sexual orientation and gender identity as barriers to trauma discussion and incorporate systematic and societal factors. |
| **19**. Pathways should be designed with recognition of the specific challenges faced by women with limited English proficiency or other communication difficulties when disclosing trauma. These challenges may include:   - Reluctance to disclose in the presence of an interpreter. It is essential to acknowledge and address potential barriers that interpreters might pose to open communication. - Fear that the interpreter will breach confidentiality and disclose sensitive information to others in the community. Strategies should be implemented to build trust and ensure interpreter confidentiality. - Reluctance to disclose in the presence of partners, family, or friends who are acting as interpreters. It is crucial to discourage this practice, emphasising the importance of neutral and professional interpreters. - Limited literacy in their own language can mean translated materials are not helpful and make women feel ashamed. Services should strive to provide accessible information such as audio translations of questionnaires and information leaflets. - Difficulty understanding technical terms, written information, or subtle nuances even for women with good conversational English. Efforts should be made to communicate information in a clear, straightforward manner to ensure understanding across varying levels of English proficiency. - Services should also consider how they can meet the needs of women who have other communication needs, including hearing difficulties, learning disabilities, neurodivergence, or low literacy.   *This recommendation aims to address the barriers faced by women with limited English proficiency in discussing previous trauma.* | Studies included in the EMPATHY systematic review highlighted the difficulties faced by women with limited English proficiency in trauma discussions, citing interpreters as a barrier to disclosure (16,23,24).    EMPATHY study interview participants universally felt that trauma discussions are more difficult for women with limited English. Participants highlighted that even women with good conversational English may struggle to understand complex information or pick up subtleties. Several participants noted that where possible it is beneficial to have consistency of interpreters and that interpreters would benefit from training in how to ask sensitive questions. Participants noted that not all women are literate in their first language, and therefore translating questionnaires into other languages might result in women not understanding the questions, possibly feeling ashamed, or inadvertently asking their abuser or neighbour to translate for them, not realising the sensitivity of the information. The idea of questionnaires which were translated into audio, rather than written form, was popular with participants. Participants pointed out that lack of literacy is not only an issue for women with limited English, and materials should be written with this in mind. Law et al. (2021) advise services to consider the needs of individuals with language or literacy difficulties. | There was unanimous agreement on the importance of adapting trauma discussions for women with limited English proficiency. Participants stressed the need for time and resources to develop these pathways and highlighted limitations in referral services for women with limited English skills. The importance of ensuring continuity of interpreters whenever possible, in addition to the choice of using an interpreter or tools like Google Translate, was also highlighted. Participants pointed out the complexities when interpreters speak different dialects, often involving intersecting class and caste issues. Furthermore, the recommendation addresses the reluctance of some women to disclose previous trauma when family or friends act as interpreters. Several participants recommended the guidance should explicitly state not to use family members as interpreters, with one arguing *‘there should be strong onus on the trust to recruit and train appropriate interpreters’* (R15). Respondents also spoke about the need to tailor support to different needs, including creating pathways and materials for people who are neurodivergent, have learning disabilities, or are deaf.  In response to feedback from the guideline consultation, this recommendation was expanded to provide detailed guidance on linguistic barriers and acknowledge other communication barriers including hearing difficulties, learning disabilities, neurodivergence, or low literacy. |
| **20.** Routine trauma discussion pathways should be tailored to local resources and services. Women should also be informed of national support organisations to ensure a minimum level of support for all women, regardless of location. It is important to acknowledge that some women prefer anonymous support options, such as telephone-based or national rather than local services, due to concerns about confidentiality and social encounters with support providers. Additionally, poverty should be recognised as a barrier to accessing support.  *This recommendation aims to leverage the expertise of voluntary sector organisations, involve experts by experience in service design, and ensure women are signposted to appropriate local and national support organisations.* | SAMHSA (2014) recommends services collaborate with community providers with expertise in delivering evidence-based trauma services. The NHS England and Improvement guide on trauma-informed care emphasises the importance of co-production of services with experts by experience, suggesting the engagement of voluntary service organisations and Maternity Voices Partnerships to aid in this (Law et al., 2021). | Respondents largely supported the recommendation that trauma discussions should be tailored to fit local resources and services, stressing the importance of care providers being informed about local support options and their accessibility. However, participants noted the need for a consistent level of support for all women, regardless of location, and noted that some women might prefer national rather than local support to maintain anonymity. Several participants highlighted the essential role of experts by experience and the voluntary sector, including respondent R9:  *‘Great to see inclusion of VCSE [Voluntary, Community and Social Enterprise] who play such a vital role and often have years of experience in this space. The impact of a good local knowledge of what's out there for women and families is gold and integral to a holistic and relational model of care.’*  In response to feedback from the guideline consultation, the recommendation was expanded to underscore the provision of a minimum level of support to all women and recognise poverty as a barrier to accessing support. |
| **21.** While respecting women’s individual rights to confidentiality and their choices regarding documentation of trauma disclosures in medical records, efforts should be made to measure the uptake and impact of routine trauma discussions. Collected data could include:   - Proportion of staff trained in conducting trauma discussions. - Proportion of women asked about previous trauma. - Basic sociodemographic information. - Number of women who disclosed trauma and types of traumas disclosed. - Changes in care resulting from trauma disclosures. - Uptake of referrals made. - Impact on related services such as referrals to mental health and addiction services. - Impact of routine trauma discussion on outcomes such as health, quality of life and experience of parenting.   In analysing the data, both the overall dataset and specific results relating to marginalised groups and individuals from different cultural backgrounds should be considered to ensure inclusivity and representation of diverse voices.  *The recommendation aims to evaluate the impact of routine trauma discussions on maternity care provision and women’s outcomes, enabling a comprehensive understanding of the intervention's effectiveness and driving quality improvement efforts.* | SAMHSA (2014) advise that organisations should identify specific measures or indicators to gauge organisational progress towards achieving a trauma-informed approach, incorporating these metrics into quality assurance processes. NHS England and NHS Improvement (Law et al., 2021) highlight the importance of evaluating trauma-informed services to establish whether they improve women’s experiences and outcomes. Proposed measures include changes in service user experiences of care, disclosure rates, and the implementation of trauma-informed approaches by staff. | Most respondents agreed on the importance of measuring trauma discussion effectiveness to understand if the care provided meets women’s needs and support further funding. However, they acknowledged challenges in accurately assessing effectiveness. For example, Participant R15 suggested that a genuine measure would be a decrease in re-traumatisation rates, highlighting the complexity of evaluating the impact of these discussions. Participants stressed the significance of collaboration between services, frontline practitioners, experts by experience, and voluntary sector organisations to develop evaluation materials and mechanisms. Some suggested creating a national framework for implementation and evaluation that could be adapted locally. Additionally, they proposed having a dedicated staff member responsible for implementing and evaluating routine trauma discussions, along with a champion at the board level.  In response to feedback from the guideline consultation, the recommendation was expanded to include the input of experts by experience and voluntary sector organisations in developing measures of effectiveness, and recommend the separate analysis of data relating to marginalised groups and women from different cultural backgrounds. |
| **22**. Feedback should be sought at a local level from women using maternity services and staff regarding routine discussion of previous trauma. The aim of this feedback is to establish whether it is acceptable and helpful, and to identify unintended consequences, such as the risk of re-traumatisation for women or negative impact on staff wellbeing. To encourage open communication and constructive criticism, feedback collection should be anonymous. Services should collaborate with voluntary service organisations to develop strategies to seek feedback from marginalised populations. Responses should be analysed both as a whole, and separately for marginalised groups and different cultural backgrounds, to ensure trauma discussions are equitable.  *This recommendation aims to assess the acceptability and effectiveness of these discussions while identifying any unintended consequences, such as re-traumatisation of women or negative impacts on staff wellbeing.* | Several studies included in the EMPATHY systematic review showed that poorly managed trauma conversations can detrimentally affect women's subsequent engagement with healthcare services (12,14,16). Additionally, five studies in the review highlighted the emotional difficulties for maternity care providers hearing trauma disclosures (4,7,8,11,21). Participants in EMPATHY study interviews echoed concerns about the potential harm arising from insensitive trauma discussions, and the impact on staff of trauma conversations. To mitigate these risks, SAMHSA guidance (2014) recommends the development of processes for soliciting feedback from both service users and staff, ensuring anonymity and confidentiality to facilitate open communication and constructive criticism. In alignment with this, the NHS England and NHS Improvement guide by Law et al. (2021) advocates for the evaluation of trauma-informed services to determine whether such services effectively improve experiences and outcomes for women. | Respondents largely supported the collection of local feedback from women using maternity services and staff about routine trauma discussions, emphasising the importance of seamlessly integrating feedback collection into normal processes to prevent it becoming an additional burden. Several participants highlighted the need for trauma-informed methods when collecting local feedback to avoid re-traumatisation. They also mentioned that the cultural barriers to discussing trauma noted in recommendation 18 could deter some women from providing feedback, potentially affecting the data. To address these concerns, respondent R15 proposed that services collaborate with dedicated charities or community-based groups experienced in addressing trauma-related issues.  As a result of feedback from the guideline consultation, the recommendation was expanded to assure anonymity for those providing feedback and consider marginalised populations in feedback collection and analysis. |
| **23**. While upholding women’s rights to confidentiality, maternity services should collaborate with each other to share findings and identify best practices. Findings should also be shared with the steering group, staff conducting trauma discussions, and local voluntary service organisations.  *This recommendation aims to promote continuous improvement of trauma discussions within maternity care services.* | The NHS England and NHS Improvement guide to implementing trauma-informed perinatal care (Law et al., 2021) advises commissioners and providers to consider how they can continually improve service design and delivery. Similarly, SAMHSA (2014) stresses the importance of quality improvement. | Respondents generally supported this recommendation, acknowledging its role in ongoing learning and the development of services. Participant R10 suggested that providing clear guidance on sharing findings while maintaining confidentiality, such as using composite narratives, would be beneficial. Additionally, participants proposed co-producing findings with survivors as a valuable approach. |
